# Modified Particle Swarm Optimization Algorithms for the Generation of Stable Structures of Carbon Clusters, C_n_ (*n* = 3–6, 10)

**DOI:** 10.3389/fchem.2019.00485

**Published:** 2019-07-12

**Authors:** Gourhari Jana, Arka Mitra, Sudip Pan, Shamik Sural, Pratim K. Chattaraj

**Affiliations:** ^1^Department of Chemistry and Centre for Theoretical Studies, Indian Institute of Technology, Kharagpur, India; ^2^Department of Electronics and Electrical Communication Engineering, Indian Institute of Technology, Kharagpur, India; ^3^Fachbereich Chemie, Philipps-Universität Marburg, Marburg, Germany; ^4^Department of Computer Science and Engineering, Indian Institute of Technology, Kharagpur, India; ^5^Department of Chemistry, Indian Institute of Technology Bombay, Mumbai, India

**Keywords:** global minimum energy structures, density functional theory, carbon clusters, particle swarm optimization, multi-threaded code, Metaheuristic Algorithm, Gaussian

## Abstract

Particle Swarm Optimization (PSO), a population based technique for stochastic search in a multidimensional space, has so far been employed successfully for solving a variety of optimization problems including many multifaceted problems, where other popular methods like steepest descent, gradient descent, conjugate gradient, Newton method, etc. do not give satisfactory results. Herein, we propose a modified PSO algorithm for unbiased global minima search by integrating with density functional theory which turns out to be superior to the other evolutionary methods such as simulated annealing, basin hopping and genetic algorithm. The present PSO code combines evolutionary algorithm with a variational optimization technique through interfacing of PSO with the Gaussian software, where the latter is used for single point energy calculation in each iteration step of PSO. Pure carbon and carbon containing systems have been of great interest for several decades due to their important role in the evolution of life as well as wide applications in various research fields. Our study shows how arbitrary and randomly generated small C_n_ clusters (*n* = 3–6, 10) can be transformed into the corresponding global minimum structure. The detailed results signify that the proposed technique is quite promising in finding the best global solution for small population size clusters.

## Introduction

Over the past decades, studies on nature-inspired swarm intelligence based meta-heuristic algorithms have become a topic of paramount interest in the allied research fields. To date, various optimization problems have been addressed using these algorithms and these have turned out to be an important tool in analyzing physical systems, in solving the complex problems and in searching for the best solution from a set of all possible feasible solutions. Particularly, global optimization (GO) has become very challenging in the development of computational fields. Search for the globally optimal solution is more crucial than that for a local optima as the former corresponds to the correct and desirable solution. Fundamentally, GO methods can be divided into two broad classes, namely (i) deterministic algorithms and (ii) stochastic algorithms. Although deterministic methods are capable of providing a guaranteed global optimum solution, the necessary properties of objective function and some constraints are required as well. On the other hand, stochastic methods can provide successful results in finding the global best solution without consideration of any assumption of differentiability and continuity of objective function. Until now, several stochastic methods such as genetic algorithms (GA) (Holland, 1992; Grüninger and Wallace, 1996; Ursem, 2000; Deb et al., 2002; Poli and Langdon, 2002; Dilettoso and Salerno, 2006; Krug et al., 2010), simulated annealing (SA) (Woodley et al., 1999; Abraham and Probert, 2006; Glass et al., 2006; Oganov and Glass, 2006; Trimarchi and Zunger, 2007), differential evolution (DE) (Storn, 1996; Storn and Price, 1997; Price et al., 2006; Rocca et al., 2011), harmony search (HS) (Geem, 2000, 2001, 2006; Geem et al., 2001, 2005; Diao and Shen, 2012; Gholizadeh and Barzegar, 2013; Hadwan et al., 2013; Manjarres et al., 2013; Nekooei et al., 2013; Wang and Li, 2013; Hoang et al., 2014; Fattahi et al., 2015; Weyland, 2015; Assad and Deep, 2016), ant colony optimization (ACO) (Colorni et al., 1992; Dorigo, 1992; Dorigo and Di Caro, 1999; Zlochin et al., 2004; Dorigo and Birattari, 2010; Korošec et al., 2012), cuckoo search (CS) (Payne and Sorensen, 2005; Yang and Deb, 2009; Inderscience, 2010), bat algorithm (BA) (Altringham et al., 1996; Richardson, 2008; Yang, 2010a,b), artificial bee colony optimization (ABC) (Karaboga and Basturk, 2007, 2008; Omkar et al., 2011; Fister and Žumer, 2012; Li G. et al., 2012), honey bee mating optimization (HBMO); (Pham et al., 2005; Haddad et al., 2006; Afshar et al., 2007; Jahanshahi and Haddad, 2008; Marinakis and Marinaki, 2009; Pham and Castellani, 2009, 2014, 2015; Bitam et al., 2010; Gavrilas et al., 2010; Marinaki et al., 2010; Chakaravarthy and Kalyani, 2015; Nasrinpour et al., 2017; Rajasekhar et al., 2017), and multi-colony bacteria foraging optimization (MC-BFA) (Chen et al., 2010) have been developed and used in various research fields including global optimization purpose. Moreover, some advanced and more promising methods are continuously being proposed including random sampling method (Pickard and Needs, 2006, 2007, 2008), minima hopping (Kirkpatrick et al., 1983; Pannetier et al., 1990), basin hopping (Nayeem et al., 1991; Wales and Doye, 1997), meta-dynamics (Martonák et al., 2003, 2005; Guangneng et al., 2005), data mining (Mujica and Needs, 1997) and Particle Swarm Optimization (PSO) (Kennedy and Eberhart, 1995, 1999; Kennedy, 1997; Shi and Eberhart, 1998; Eberhart and Shi, 2001; Li, 2007; Özcan and Yilmaz, 2007; Poli, 2007, 2008; Barrera and Coello, 2009; Li M. et al., 2012; Qu et al., 2012; Bonyadi and Michalewicz, 2017), modified PSO (Zheng et al., 2007), adaptive particle swarm optimization (APSO) (Zhan et al., 2009), multi-dimensional PSO for dynamic environments (Zhi-Jie et al., 2009; Kiranyaz et al., 2011; Bhushan and Pillai, 2013), which indeed show different numerical performances.

Out of these numerous techniques, PSO is a very renowned iterative process which works intelligently by utilizing the concept of exploring and exploiting together in the multidimensional search space for finding optimal or near-optimal solutions. The learning strategies of this technique for the searching of structural information are very much suitable and reliable in an active area of GO research. This evolutionary computational method was first invented by Kennedy and Eberhart (1995) and Kennedy (1997) in the mid 1990s on graceful collaborative motion of biological populations rooted on the concept of “information sharing and collective intelligence.” This adaptive metahurestic technique emphasizes on overcoming the energy barriers, particularly by the upgradation of positions and velocities following the individual or personal best which again follows the global best one. After several developments (Reeves, 1983; Reynolds, 1987; Heppner and Grenander, 1990; Millonas, 1993; Clerc, 1999; Eberhart and Shi, 2000; Banks et al., 2007; Bui et al., 2007; Khan and Sadeequllah, 2010), adaptation (Wang et al., 2011), modifications (like niching with PSO Brits et al., 2002; Engelbrecht and Van Loggerenberg, 2007; Sun et al., 2007; Nickabadi et al., 2008; Wang J. et al., 2009; Wang Y. et al., 2009) single solution PSO (Liu and Wang, 2006; AlRashidi and El-Hawary, 2007; Li and Li, 2007; Liu B. et al., 2007; Liu D. et al., 2007; Petalas et al., 2007; Schutze et al., 2007; Zhang et al., 2007; Zhang and Wang, 2008; Benameur et al., 2009) and multi-objective optimization (Cai et al., 2004, 2009; Call et al., 2007; Chandrasekaran et al., 2007; Abido, 2009; Alatas and Akin, 2009; Dehuri and Cho, 2009; De Carvalho et al., 2010; Goh et al., 2010; Briza and Naval, 2011; Chen et al., 2011), constraint optimization with PSO (Cao et al., 2004; AlRashidi and El-Hawary, 2006; Sun and Gao, 2008; Ma et al., 2009; Sivasubramani and Swarup, 2009), discrete PSO (Yin, 2004; Yeh, 2009; Yeh et al., 2009; Unler and Murat, 2010), dynamic environment of PSO (Shao et al., 2004, 2008; Zhang et al., 2006; Chen et al., 2007; Liu X. et al., 2007; Yang et al., 2007; Du and Li, 2008; Wang and Xing, 2008; Zhao et al., 2008; Cheng et al., 2009; Wang Y. et al., 2009; Bae et al., 2010) and parameterization (Eberhart and Shi, 2001; Shi, 2001; Trelea, 2003; Li-Ping et al., 2005; Talbi, 2009; Pedersen, 2010; Bansal et al., 2011) on the original PSO, more recently global optimization of small boron clusters (B_5_ and B_6_) using a more advanced PSO approach has been reported with great success (Mitikiri et al., 2018).

On the other hand, the investigation on pure carbon molecules existing in various structural forms (chains/cyclic rings) has been a matter of great interest in the research area of organic, inorganic and physical chemistry (Weltner and Van Zee, 1989) as the study and production of carbon-riched molecules in the laboratory are notoriously difficult due to their high reactivity and transient like behavior. They are also very important in astrophysics, particularly in connection with the chemistry of carbon stars (Bernath et al., 1989), comets (Douglas, 1951), and interstellar molecular clouds (Bettens and Herbst, 1997). Long carbon chains are also believed to act as carriers of diffuse interstellar bands (Fulara et al., 1993). Moreover, carbon clusters are also important constituents in hydrocarbon flames and other soot-forming systems (Kroto and McKay, 1988) and they play an important role in gas-phase carbon chemistry where they serve as intermediates for the production of fullerenes, carbon tubes, thin diamond and silicon carbide films (Koinuma et al., 1996; Van Orden and Saykally, 1998). Therefore, the study about the structures and stabilities of carbon clusters is very important to thoroughly understand the complex chemical environment of such systems and also to shed light into the remarkable bonding capability of carbon which is able to form single, double and triple bonds. They together make the study on the structural information of carbon clusters in the field of theoretical research a subject of immense interest and it started before the development of fullerene chemistry (Pitzer and Clementi, 1959; Weltner and Van Zee, 1989; Martin et al., 1993; Hutter et al., 1994).

Due to the reduction in angle strain, carbon clusters larger than C_10_ are likely to exist as monocyclic rings, while smaller ones possess low-energy linear structures. Moreover, it was reported that for small clusters with even number of carbon atoms such as C_4_, C_6_, and C_8_, the cyclic form is either the lowest energy isomer or almost isoenergetic to their linear counterparts (Raghavachari and Binkley, 1987; Watts et al., 1992; Hutter and Lüthi, 1994; Pless et al., 1994; Martin and Taylor, 1996). In this study, we have checked the efficiency of our newly developed multi-threaded PSO code, written in python, and augmented by Gaussian 09 program package (Frisch et al., 2013) to locate global minimum energy structures for C_n_ clusters (*n* = 3–6). Particularly, we want to test our code for the system where two minima are located at two deep well points on the PES as in the case of C_6_ cluster. We kept the cluster size small in order to compare the performance of our code to other popular evolutionary simulation techniques such as SA, GA, and BH.

## Currently Proposed and Implemented PSO Technique

Initially, random structures are generated within certain range (−3, 3) in a multidimensional search space followed by upgradation of velocity and position vectors through swarm intelligence. After completion of every iteration, energy of each particle is calculated and a convergence criterion is verified with the help of the Gaussian 09 package interfaced with the present PSO algorithm. Individual best and global best positions are updated. If the energy values of successive 30 iterations remain same, the program automatically terminates. Finally a new set of initial structures are generated from the related output structures and the process is continued till the self-consistency is achieved.

In order to check the efficiency of our proposed PSO method over some most familiar GO methods like advanced BH, SA, and GA methods, the results for C_5_ cluster have been analyzed, as a reference system.

## A Comparative Account of the Current PSO Method with Other Existing Approaches

We have made the computer experiment to compare our proposed PSO with the other popular evolutionary simulation techniques such as SA, GA and advanced BH.

### Comparison of Performances of PSO and GA

- The most important distinction between our proposed DFT-PSO with GA is the sharing of information. In GA, chromosomes share information with each other, whereas in PSO the best particle informs the others and the information of variables is stored in small memory. Again, PSO search for the global best solution is unidirectional, while GA follows the parallel searching process.
- In contrast to GA, PSO does not use any genetic kind of operator, i.e., crossover and mutation, and the internal velocity leads the particle to the next better place.
- PSO implementation is more simple and easier than GA as it deals with few parameters (like position and velocity only).
- GA provides satisfactory results in case of combinatorial problems, PSO being less suitable there.
- PSO takes much less time to execute and the convergence rate is also faster than that of GA.

A previous study by Hassan et al. (2005) has been further recommended for more clarity and reliability of the efficiency of PSO over GA.

### Comparison of Performances of PSO and SA

In SA technique, a small perturbation is given to cluster entity at each successive step, and energy estimation is carried out consecutively. Acceptance of perturbation depends on the obtained energy value. If the obtained energy is better than the previous one, the perturbation as well as the move with low cost is accepted. Otherwise, the process excludes it and the Boltzmann probability distribution is applied at a given temperature. The particle (individual cluster) in SA takes much time to generate different lower energy structures. The temperature decreases during the whole course of the process very slowly and at the end of the run it attains the least value. In contrast, such kind of perturbation or temperature variable is not present in PSO. Both exploitation and exploration techniques drive the particle in PSO, while only exploitation is used in SA. So, there are more chances to trap the particles in local minima in case of SA being a single-based technique than PSO. On the other hand, PSO, being the population based technique, is able to swarm wherever (different places of mountain or lower point of valleys) be the particle in the search space.

### Comparison With Basin Hopping

Wales and Doye jointly described basin hopping algorithm (Berg and Neuhaus, 1991; Wales and Doye, 1997; Doye et al., 1998) which has become a popular stochastic search process to find out the desired global best solution of an object function. This method is basically a Monte Carlo technique, which works in a perturbative and iterative manner. At first, a random coordinate of a particle is considered. Then, random perturbation is applied to the configuration considering the fact that the particle remains in a local basin which is then followed by the minimization of energy functional to get a better solution. Energy estimation is again carried out and the process is repeated until the best configuration or the lowest energy structure is achieved. The most important thing is that the applied perturbation should be large enough to get out of a local basin.

## Algorithm and Computational Details

At the beginning, a set of random coordinates of C_n_ clusters (particles) with random positions and velocities are considered. The newer sets of coordinates are updated through PSO run to find out global best position or configuration. The local best configuration (p_*best*_) or that having the lowest energy value obtained locally so far is stored in a small memory variable which is then followed by the searching of global best (g_*best*_) configuration (among the set of p_*best*_) through an exploration technique. Ultimately, the best optimal solution is achieved.

The new velocities ($v_{\text{i}}^{\text{t}+1}$) and positions ($x_{\text{i}}^{\text{t}+1}$) of particles in ith generation obey the following equations where $x_{\text{i}}^{\text{t}}$ and $v_{\text{i}}^{\text{t}}$ are the current position and velocity.

(1) $$\begin{array}{rc} v_{\text{i}}^{\text{t}+1}= & w*v_{\text{i}}^{\text{t}}+d_{1}*\varepsilon_{1}*\left(p_{best}-x_{\text{i}}^{\text{t}}\right)+d_{2}*\varepsilon_{2}*\left(g_{best}-x_{\text{i}}^{\text{t}}\right) \end{array}$$

(2) $$\begin{array}{rcl} x_{\text{i}}^{\text{t}+1} & = & x_{\text{i}}^{\text{t}}+v_{\text{i}}^{\text{t}+1} \end{array}$$

ε_1_ and ε_2_ are chosen randomly in between (0,1). The tendency of a particle to remain in its current position is called inertia coefficient denoted by w. d_1_ and d_2_ (which can be modified as per requirement) which are referred to as individual coefficient of acceleration and global coefficient of acceleration, respectively. These two coefficients guide the particles to meet convergence so that all the candidate solutions in the problem space efficiently achieve the global minimum (see Table 1).

**Table 1:** PSO Parameters.

**Parameters**	**Value**
Population (*N_*pop*_*)	10
Inertia Coefficient (*w*)	0.4–0.8
Individual coefficient of acceleration (d_1_)	2
Global coefficient of acceleration (d_2_)	2
Random Coefficients (ε_1_; ε_2_)	[0,1]

After the completion of each PSO run, optimization of global best structural units of C_n_ clusters (*n* = 3–6) are performed at the B3LYP (Lee et al., 1988; Becke, 1993)/6-311+G^*^ level in the Gaussian 09 program.

**Figure 1:**
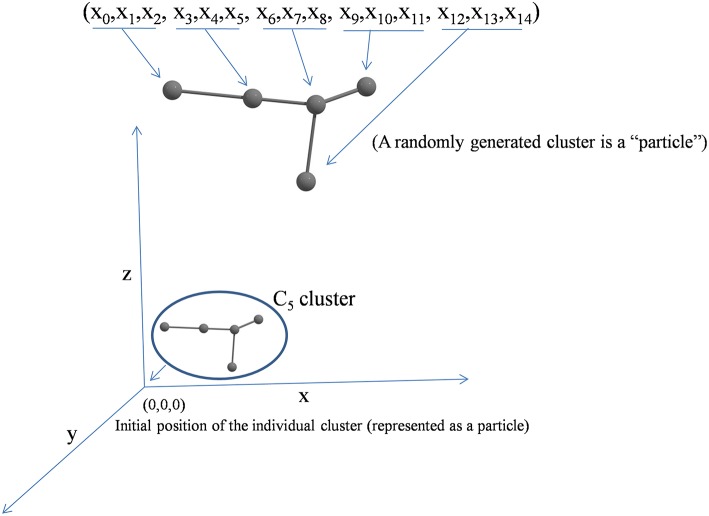
A schematic representation of a cluster in multidimensional search space.

Each randomly generated cluster unit is considered as a particle. In Figure 1 (x_0_, x_1_, x_2_,…x_3n−1_), particle comprises n atoms. Here, the coordinates of ith atom are (x_3i_, x_3i+1_, x_3i+2_).

## Parallel Implementation

One of the major advantages of using PSO as proposed in this paper over some of the classical optimization techniques is its parallelizability. The same implementation of the algorithm can be executed on machines having single core (serial implementation) or ones with multiple cores (laboratory grade clusters) or high performance computing (HPC) systems. Changing a couple of header parameters in the program is sufficient to make it portable across a wide range of platforms. We have tested both a serial as well as a parallel implementation of our programs. Results on parallel implementation are reported. It may be noted that our PSO algorithm implemented in Python invokes the Gaussian software as a system call. Each such parallel call, one for each particle of the PSO algorithm, causes a new instance of Gaussian to be executed. The number of cores on which each Gaussian instance runs is dependent on the available number of processor cores. However, at the end of every iteration, PSO has to recompute the best and global best positions of individual particle before updating the velocity values from which the new positions of the particles are determined. These are done by reading the output log files generated by Gaussian for each particle. This implies that the results of all the parallel invocations of Gaussian need to be completed before the iteration-end processing can be done. We have implemented appropriate synchronization mechanisms to enable such parallel implementation and hence, the code base is portable across multiple platforms.

## Computational Setup

All our computations were carried out on a single server having two Intel 2.70 GHz Xeon E5-2697 v2 processors and 256 GB of RAM. Each processor has 12 cores. Leaving aside a few cores for operating system and other housekeeping processes, we made use of 30 threads for executing our PSO algorithm. A PSO population size of 15 particles implies that 2 threads could be used for each instance of Gaussian. Also, 8 GB of RAM was dedicated to each such instance. As mentioned before, the number of PSO particles, RAM assignment and the number of threads for each Gaussian call are set as input hyper parameters. The completely parameterized implementation of PSO has been done in Python invoking Gaussian for energy calculation in a multi-threaded environment. This is one of the unique features of our work, which has not yet been reported in the literature for stable structure prediction of C_n_, to the best of our knowledge.

## Results and Discussion

In our study, each C_n_ cluster unit (each individual unit) is considered to be a swarm particle in a multidimensional potential energy surface (PES) where the stationary points (maxima, minima, and higher order saddle points) are connected. The randomly generated individual particle is governed by a position vector and a velocity vector. Again, each position vector representing a candidate solution in the hyperspace starts searching for the optima of a given function of several variables by updating generations in iterative process without much of any assumption leading to a minimum energy structure. After iteration the particle driven by a velocity vector changes its search direction. The position and velocity vectors together store the information regarding its own best position or the local best position (called p_best_) seen so far and a global best position (called g_best_) which is obtained by communicating with its nearest neighbors. Further, the advancement of particles toward the global best position is attained via particle swarm optimizer ideology and they gravitate toward the global best solution with the help of the best variable memory values. Our proposed PSO implementation explores rapidly without being entrapped in local optima and executes extensively, followed by immediate convergence to the desired objective value, the global optima.

The results of global optimization of C_n_ clusters (*n* = 3–6, 10) considering a maximum of 1,000 runs starting from the random choices of input configuration are shown in Table 2. The global stable structure (best solution) can be obtained by fulfilling the termination criteria along the convex function of the information matrix when one of the particles reaches the target. Initially, 10 different random configurations have been chosen by setting random initial positions and velocities of all particles followed by the Gaussian interfaced PSO driven operation to get the global optimum structure (see Table 2).

**Table 2:** The randomly chosen 10 different molecular frameworks of C_n_ (*n* = 3–6, 10) with singlet and triplet spin multiplicity converge to the global minimum energy structures (Bond lengths are given in Å unit and the relative energies, ΔE w.r.t the global minimum energy structures in brackets are given in kcal/mol).

**Clusters**	**Initial structure**	**Final structure using PSO**	**Final optimized energy (bond lengths)**
C_3_ cluster	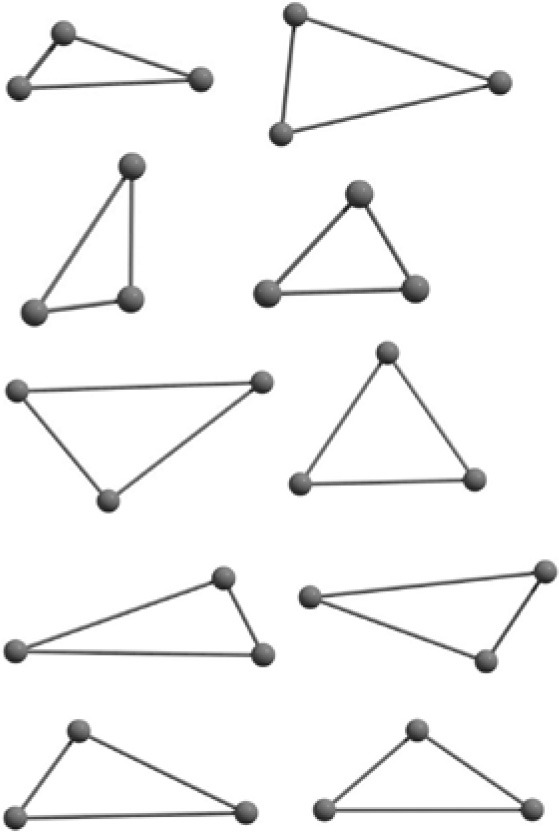	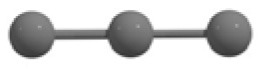	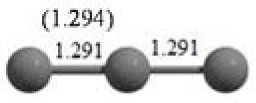 *D*_∞*h*_, S (E = −114.0769 a.u.)
C_4_ cluster	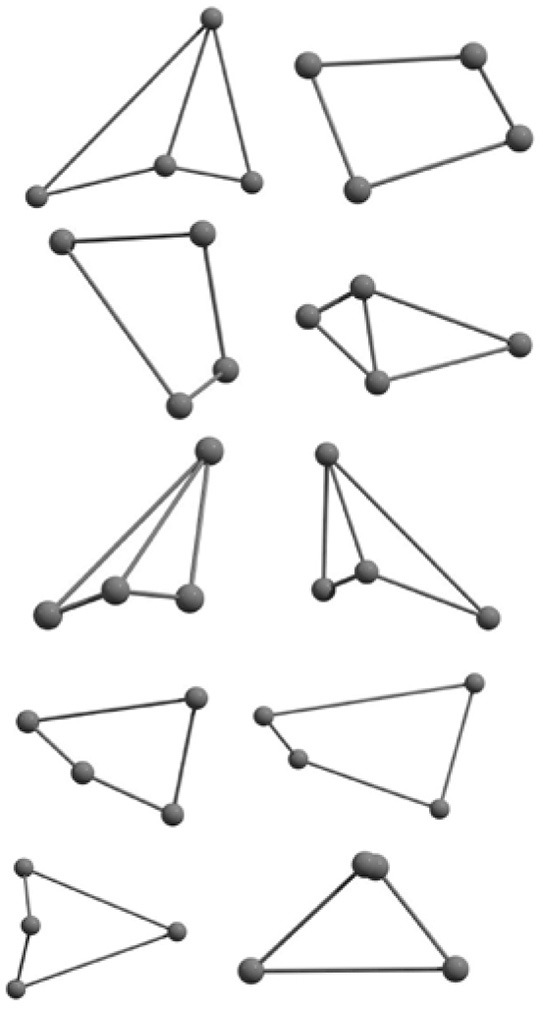	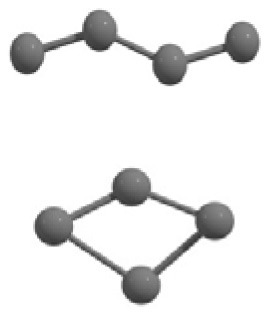	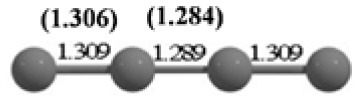 *D*_∞*h*_, T E = −152.1320 a.u. [0.0] 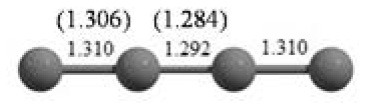 *D*_∞*h*_, S E = −152.1036 a.u. [17.8] 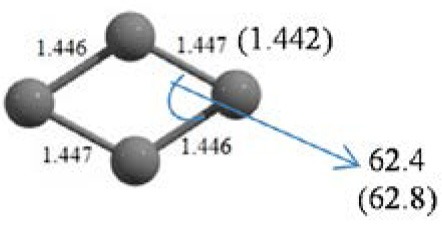 *D*_2*h*_, S E = −152.1062 a.u. [16.2]
C_5_ cluster	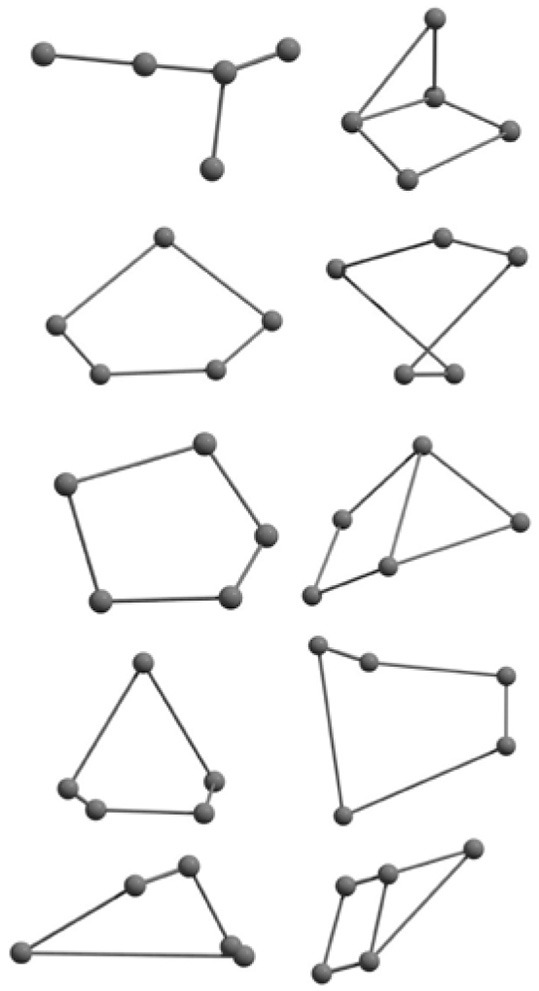	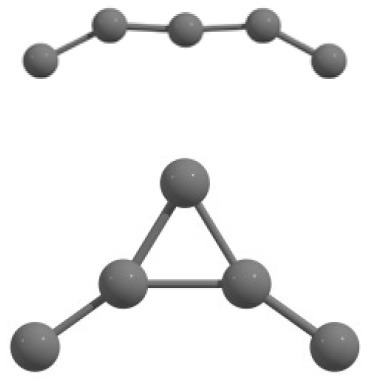	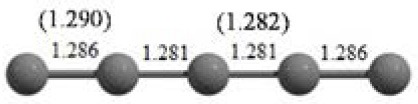 *D*_∞*h*_, S E = −190.2546 a.u. [0.0] 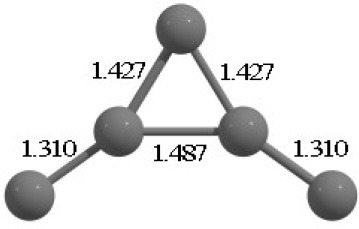 *C*_2*v*_, S E = −190.1350 a.u. [75.1]
C_6_ cluster	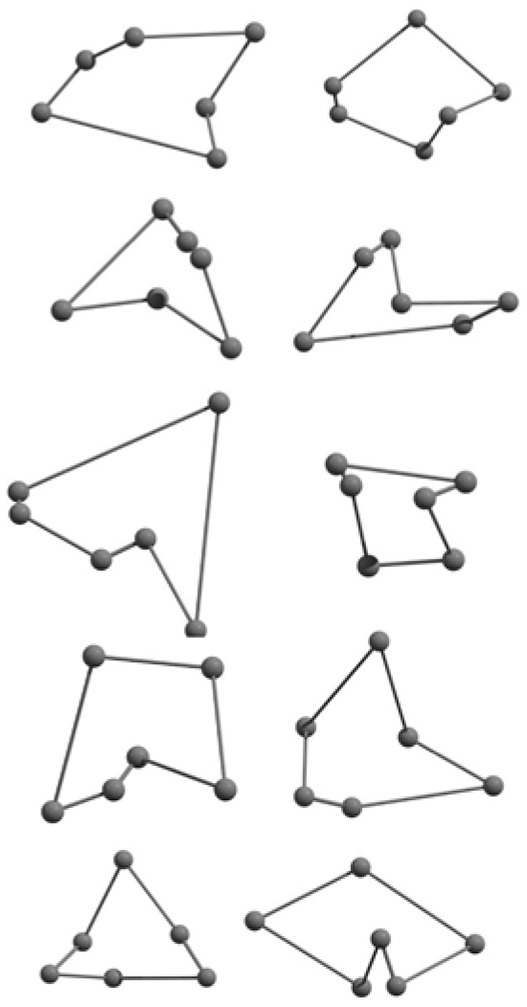	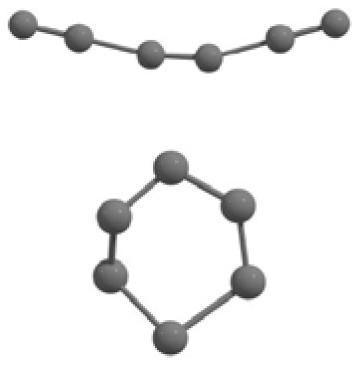	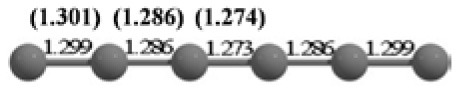 *D*_∞*h*_, T E = −228.3181 a.u. [0.0] 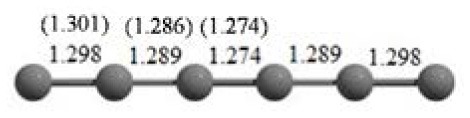 *D*_∞*h*_, S E = −228.2969 a.u. [13.3] 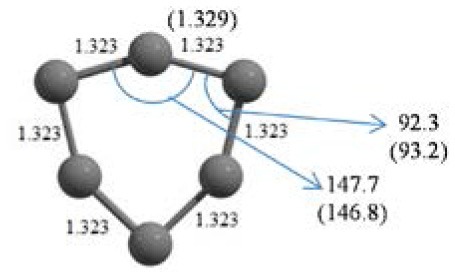 *D*_3*h*_, S E = −228.3071 a.u. [6.9]
C_10_ cluster	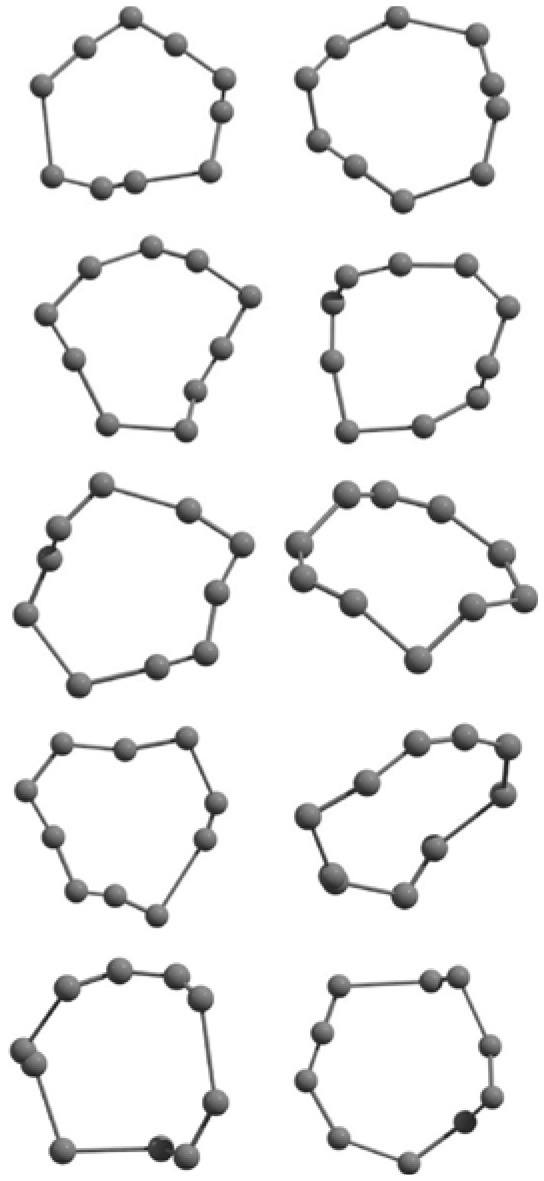	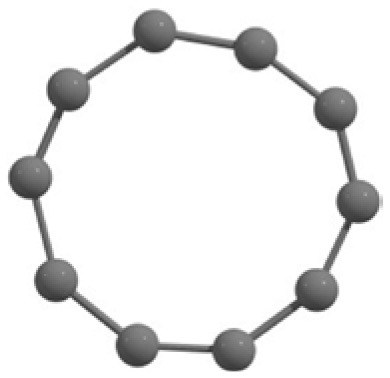	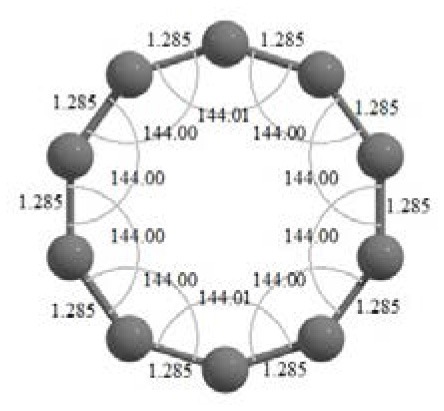 *D*_10*h*_, S (E = −380.7543 a.u.)

*All coordinates are provided in Supporting Information. (Experimental bond lengths and angles are provided within the parenthesis in the final optimized structure)*.

It is a very fascinating aspect that Gaussian optimization technique works in such a way that the guess structure can be stuck at local minima which may or may not be the global minimum. But, it is obvious that our proposed modified PSO implementation converges to the most stable structure where all the particles exist in a given range in the multidimensional hyperspace. However, sometimes atoms of the randomly generated particles (each individual cluster unit) are not in the limit of bonding perception and they might overlap on each other. In order to understand whether the atoms remain in the same molecular framework or not, we have connected the randomly deployed particles with solid lines in the following figures and they do not necessarily imply true bonds (see Table 2). In case of C_3_ cluster, the structure obtained after the end of the PSO run (linear, *D*_∞*h*_ point group) exactly matches with the structure obtained after the final G09 optimization in terms of bond length and energy. C_5_ cluster also shows linear geometry with *D*_∞*h*_ point group and singlet electronic state after final optimization step. A significantly higher energy cyclic isomer is also found in this case. On the other hand, C_4_ and C_6_ clusters (containing even number of C atoms) give both linear (*D*_∞*h*_) and ring structures (*D*_2*h*_ for C_4_ and *D*_3*h*_ for C_6_). Corresponding energies and bond lengths are provided in Table 2. The computed geometrical parameters and minimum energy structures match excellently with the previously reported experimental results (Raghavachari and Binkley, 1987; Watts et al., 1992; Hutter and Lüthi, 1994; Pless et al., 1994; Martin and Taylor, 1996; Van Orden and Saykally, 1998). For both C_4_ and C_6_ clusters, the lowest energy isomer has linear form in triplet state, whereas the linear singlet state is 17.8 (C_4_) and 13.3 (C_6_) kcal/mol higher in energy than the corresponding triplet forms. In addition to the small cluster systems, we have also checked the efficiency and the robustness of our implemented PSO code to find the global minimum for a relatively larger sized cluster, C_10_. The results show that the present code can successfully locate the desired *D*_10*h*_ symmetric ring structure which is the most stable isomer in this case.

In the present context, we have also carried out DFT-SA and DFT-BH methods considering same object energy function as in our proposed PSO method to compare the obtained results (see Table 3). The tabulated values clearly reflect that the present PSO method is superior to other methods based on the time to locate the GM, energy values after completion of all runs of the studied methods and the number of iteration steps needed to get the final structure.

**Table 3:** Comparison of PSO results with other more popular evolutionary GO techniques as applied to the C_5_ cluster starting from the corresponding local minima structures.

**Comparison in terms of**	**Advanced basin hopping (BH)**	**Simulated annealing** **(SA)**	**Modified PSO**
Execution time to locate the global minimum (GM)	305,140 s	12,959 s	8,898 s
Energy of the global minimum (Energy after completion of iterations)	−190.2546 a.u. (−190.2460 a.u.)	−190.2546 a.u. (−189.5141 a.u.)	−190.2546 a.u. (−190.2436 a.u.)
Number of iterations needed to get a structure close to GM	1,703 (converged)	92 (not converged)	331 (converged)

**Figure 2:**
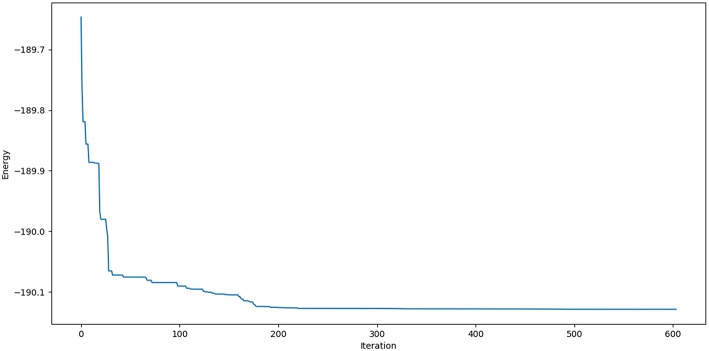
Single point energy evolution landscape of C_5_ cluster during each generation of convergence at the B3LYP/6-311+G* level.

A representative plot of C_5_ cluster (as reference) is given below to ensure the fulfillment of convergence criteria up to 600 iteration steps (see Figure 2).

## Conclusion

This systematic study for the searching of the most stable carbon based small clusters describes the effectiveness of the application of our proposed PSO technique. Currently employed less expensive and relatively less complicated computational method generates a vast potential search space depending only on the position and velocity variables. Our proposed method opens a new vista to find out global minimum energy structures effectively and accurately within a given multidimensional configuration search space. PSO implementation without much of any assumption like constraints of symmetry and externally imposed factors like temperature, pressure, etc. performs suitably and converges to a single configuration that presumably is a global minimum energy structure or may exactly fit it after Gaussian optimization. PSO can be used as a fast post-processing technique to get a global minimum or close to global minimum structure. In fact, in this study we have introduced a new easy implementation and computationally less expensive approach for the reduction of iteration steps to obtain global best configurations of small carbon clusters with exact energy values.

## Data Availability

All datasets generated for this study are included in the manuscript and/or the Supplementary Files.

## Author Contributions

GJ: interfacing with the Gaussian software, writing first version of the full manuscript, generation of the TOC, and analysis of the results. AM: implementation of the PSO algorithm in python including coding and parallelization. SP: revision of the draft manuscript and interpretation of data. SS: revision of the draft manuscript and checking of implemented code. PC: formulation of the problem, critical revision of the manuscript, and data interpretation.

### Conflict of Interest Statement

The authors declare that the research was conducted in the absence of any commercial or financial relationships that could be construed as a potential conflict of interest.

## References

[B1] Abido M. (2009). Multiobjective particle swarm optimization for environmental/economic dispatch problem. Electr. Pow. Syst. Res. 79, 1105–1113. 10.1016/j.epsr.2009.02.005

[B2] Abraham N. L. Probert M. I. (2006). A periodic genetic algorithm with real-space representation for crystal structure and polymorph prediction. Phys. Rev. B 73:224104 10.1103/PhysRevB.73.224104

[B3] Afshar A. Haddad O. B. Mariño M. A. Adams B. J. (2007). Honey-bee mating optimization (HBMO) algorithm for optimal reservoir operation. J. Franklin Inst. 344, 452–462. 10.1016/j.jfranklin.2006.06.001

[B4] Alatas B. Akin E. (2009). Multi-objective rule mining using a chaotic particle swarm optimization algorithm. Knowl Based Syst. 22, 455–460. 10.1016/j.knosys.2009.06.004

[B5] AlRashidi M. El-Hawary M. (2007). Hybrid particle swarm optimization approach for solving the discrete OPF problem considering the valve loading effects. IEEE Trans. Power Syst. 22, 2030–2038. 10.1109/TPWRS.2007.907375

[B6] AlRashidi M. R. El-Hawary M. E. (2006). Emission-economic dispatch using a novel constraint handling particle swarm optimization strategy, in Electrical and Computer Engineering, CCECE'06. Canadian Conference on IEEE (Ottawa, ON: IEEE), 664–669.

[B7] Altringham J. McOwat T. Hammond L. (1996). Bats: Biology and Behaviour. New York, NY: Oxford University Press.

[B8] Assad A. Deep K. (2016). Applications of harmony search algorithm in data mining: a survey, in Proceedings of Fifth International Conference on Soft Computing for Problem Solving (Singapore: Springer), 863–874.

[B9] Bae C. Yeh W.-C. Chung Y. Y. Liu S.-L. (2010). Feature selection with intelligent dynamic swarm and rough set. Expert Syst. Appl. 37, 7026–7032. 10.1016/j.eswa.2010.03.016

[B10] Banks A. Vincent J. Anyakoha C. (2007). A review of particle swarm optimization. Part I: background and development. Nat. Comput. 6, 467–484. 10.1007/s11047-007-9049-5

[B11] Bansal J. C. Singh P. Saraswat M. Verma A. Jadon S. S. Abraham A. (2011). Inertia weight strategies in particle swarm optimization, in Nature and Biologically Inspired Computing (NaBIC), Third World Congress on IEEE (Salamanca: IEEE), 633–640.

[B12] Barrera J. Coello C. C. A. (2009). A particle swarm optimization method for multimodal optimization based on electrostatic interaction, in 8th Mexican International Conference on Artificial Intelligence, MICAI 2009: Advances in Artificial Intelligence, MICAI 2009, Lecture Notes in Computer Science, Vol. 5845, eds Aguirre A. H. Borja R. M. Garci C. A. R.? (Berlin; Heidelberg: Springer), 622–632. 10.1007/978-3-642-05258-3_55

[B13] Becke A. D. (1993). Density-functional thermochemistry. III. The role of exact exchange. J. Chem. Phys. 98, 5648–5652. 10.1063/1.464913

[B14] Benameur L. Alami J. El Imrani A. (2009). A new hybrid particle swarm optimization algorithm for handling multiobjective problem using fuzzy clustering technique, in 2009 International Conference on Computational Intelligence, Modelling and Simulation (Brno: IEEE), 48–53. 10.1109/CSSim.2009.42

[B15] Berg B. A. Neuhaus T. (1991). Multicanonical algorithms for first order phase transitions. Phys. Lett. B 267, 249–253. 10.1016/0370-2693(91)91256-U 10045099

[B16] Bernath P. F. Hinkle K. H. Keady J. J. (1989). Detection of C5 in the circumstellar shell of IRC+ 10216. Science 244, 562–564. 10.1126/science.244.4904.562 17769400

[B17] Bettens R. Herbst E. (1997). The formation of large hydrocarbons and carbon clusters in dense interstellar clouds. Astrophys. J. 478:585 10.1086/303834

[B18] Bhushan B. Pillai S. S. (2013). Particle swarm optimization and firefly algorithm: performance analysis, in 2013 3rd IEEE International Advance Computing Conference (Ghaziabad: IEEE).

[B19] Bitam S. Batouche M. Talbi E.-G. (2010). A survey on bee colony algorithms, in IEEE International Symposium on Parallel & Distributed Processing, Workshops and Phd Forum (IPDPSW) (Atlanta, GA: IEEE), 1–8.

[B20] Bonyadi M. R. Michalewicz Z. (2017). Particle Swarm Optimization for Single Objective Continuous Space Problems: A Review. MIT Press, 25, 1–54. 10.1162/EVCO_r_00180 26953883

[B21] Brits R. Engelbrecht A. P. Van den Bergh F. (2002). A niching particle swarm optimizer, in Proceedings of the 4th Asia-Pacific Conference on Simulated Evolution and Learning (Singapore: Orchid Country Club), 692–696.

[B22] Briza A. C. Naval P. C. Jr. (2011). Stock trading system based on the multi-objective particle swarm optimization of technical indicators on end-of-day market data. Appl. Soft Comput. 11, 1191–1201. 10.1016/j.asoc.2010.02.017

[B23] Bui L. T. Soliman O. Abbass H. (2007). A modified strategy for the constriction factor in particle swarm optimization, in Progress in Artificial Life. ACAL 2007. Lecture Notes in Computer Science, Vol. 4828, eds Randall M. Abbass H. A. Wiles J. (Berlin; Heidelberg: Springer), 333–344. 10.1007/978-3-540-76931-6_29

[B24] Cai J. Ma X. Li Q. Li L. Peng H. (2009). A multi-objective chaotic particle swarm optimization for environmental/economic dispatch. Energy Convers. Manag. 50, 1318–1325. 10.1016/j.enconman.2009.01.013

[B25] Cai W. Shao N. Shao X. Pan Z. (2004). Structural analysis of carbon clusters by using a global optimization algorithm with Brenner potential. J. Mol. Struct. Theochem 678, 113–122. 10.1016/j.theochem.2004.03.017

[B26] Call S. T. Zubarev D. Y. Boldyrev A. I. (2007). Global minimum structure searches via particle swarm optimization. J. Comput. Chem. 28, 1177–1186. 10.1002/jcc.20621 17299774

[B27] Cao C.-H. Li W.-H. Zhang Y.-J. Yi R.-Q. (2004). The geometric constraint solving based on memory particle swarm algorithm, in Machine Learning and Cybernetics, 2004. Proceedings of International Conference on: IEEE (Shanghai: IEEE), 2134–2139.

[B28] Chakaravarthy T. Kalyani K. (2015). A brief survey of honey bee mating optimization algorithm to efficient data clustering. Indian J. Sci. Technol. 8:24 10.17485/ijst/2015/v8i24/59219

[B29] Chandrasekaran S. Ponnambalam S. Suresh R. Vijayakumar N. (2007). Multi-objective particle swarm optimization algorithm for scheduling in flowshops to minimize makespan, total flowtime and completion time variance, in Evolutionary Computation, 2007. CEC 2007 (Singapore: IEEE Congress), 4012–4018.

[B30] Chen H. Zhu Y. Hu K. (2010). Multi-colony bacteria foraging optimization with cell-to-cell communication for RFID network planning. Appl. Soft Comput. 10, 539–547. 10.1016/j.asoc.2009.08.023

[B31] Chen H. Zhu Y. Hu K. Ku T. (2011). RFID network planning using a multi-swarm optimizer. J. Netw. Comput. Appl. 34, 888–901. 10.1016/j.jnca.2010.04.004

[B32] Chen Y.-P. Peng W.-C. Jian M.-C. (2007). Particle swarm optimization with recombination and dynamic linkage discovery. IEEE Trans. Syst. Man Cybern. B 37, 1460–1470. 10.1109/TSMCB.2007.904019 18179066

[B33] Cheng C.-T. Liao S.-L. Tang Z.-T. Zhao M.-Y. (2009). Comparison of particle swarm optimization and dynamic programming for large scale hydro unit load dispatch. Energy Convers. Manag. 50, 3007–3014. 10.1016/j.enconman.2009.07.020

[B34] Clerc M. (1999). The swarm and the queen: towards a deterministic and adaptive particle swarm optimization, in Evolutionary Computation, 1999. CEC 99. Proceedings of the 1999 Congress on: IEEE (Washington, DC: IEEE), 1951–1957.

[B35] Colorni A. Dorigo M. Maniezzo V. (1992). Distributed optimization by ant colonies, in Proceedings of the First European Conference on Artificial Life (Cambridge, MA), 134–142.

[B36] De Carvalho A. B. Pozo A. Vergilio S. R. (2010). A symbolic fault-prediction model based on multiobjective particle swarm optimization. J. Syst. Softw. 83, 868–882. 10.1016/j.jss.2009.12.023

[B37] Deb K. Pratap A. Agarwal S. Meyarivan T. (2002). A fast and elitist multiobjective genetic algorithm: NSGA-II. IEEE Trans. Evol. Comput. 6, 182–197. 10.1109/4235.996017

[B38] Dehuri S. Cho S.-B. (2009). Multi-criterion Pareto based particle swarm optimized polynomial neural network for classification: a review and state-of-the-art. Comput. Sci. Rev. 3, 19–40. 10.1016/j.cosrev.2008.11.002

[B39] Diao R. Shen Q. (2012). Feature selection with harmony search. IEEE Trans. Syst. Man Cybern. B 42, 1509–1523. 10.1109/TSMCB.2012.2193613 22645272

[B40] Dilettoso E. Salerno N. (2006). A self-adaptive niching genetic algorithm for multimodal optimization of electromagnetic devices. IEEE Trans. Magn. 42, 1203–1206. 10.1109/TMAG.2006.871672

[B41] Dorigo M. (1992). Optimization, learning and natural algorithms (Ph.D. Thesis). Politecnico diMilano, Italy.

[B42] Dorigo M. Birattari M. (2010). Ant colony optimization, in Encyclopedia of Machine Learning, eds Sammut C. Webb G. I. (Boston, MA: Springer). 10.1007/978-0-387-30164-8

[B43] Dorigo M. Di Caro G. (1999). The ant colony optimization meta-heuristic, in New Ideas in Optimization, eds Corne D. Dorigo M. Glover F. (London: McGraw Hill, 11–32.

[B44] Douglas A. (1951). Laboratory studies of the lambda 4050 group of cometary spectra. Astrophys. J. 114:466 10.1086/145486

[B45] Doye J. P. Wales D. J. Miller M. A. (1998). Thermodynamics and the global optimization of Lennard-Jones clusters. J. Chem. Phys. 109, 8143–8153. 10.1063/1.477477

[B46] Du W. Li B. (2008). Multi-strategy ensemble particle swarm optimization for dynamic optimization. Inf. Sci. 178, 3096–3109. 10.1016/j.ins.2008.01.020

[B47] Eberhart R. C. Shi Y. (2000). Comparing inertia weights and constriction factors in particle swarm optimization, in Evolutionary Computation, 2000. Proceedings of the 2000 Congress on: IEEE (La Jolla, CA: IEEE), 84–88.

[B48] Eberhart R. C. Shi Y. (2001). Tracking and optimizing dynamic systems with particle swarms, in Evolutionary Computation, Proceedings of the 2001 Congress on: IEEE (Seoul: IEEE), 94–100.

[B49] Engelbrecht A. P. Van Loggerenberg L. (2007). Evolutionary computation, in CEC (Singapore: IEEE Congress), 2297–2302. 10.1109/CEC.2007.4424757

[B50] Fattahi H. Gholami A. Amiribakhtiar M. S. Moradi S. (2015). Estimation of asphaltene precipitation from titration data: a hybrid support vector regression with harmony search. Neural. Comput. Appl. 26, 789–798. 10.1007/s00521-014-1766-y

[B51] Fister I. Žumer J. B. (2012). Memetic artificial bee colony algorithm for large-scale global optimization, in 2012 IEEE Congress on Evolutionary Computation (Brisbane, QLD: IEEE), 1–8.

[B52] Frisch M. J. Trucks G. W. Schlegel H. B. Scuseria G. E. Robb M. A. Cheeseman J. R. (2013). Gaussian 09, Revision D.01. Wallingford, CT: Gaussian, Inc.

[B53] Fulara J. Lessen D. Freivogel P. Maier J. (1993). Laboratory evidence for highly unsaturated hydrocarbons as carriers of some of the diffuse interstellar bands. Nature 366:439 10.1038/366439a0

[B54] Gavrilas M. Gavrilas G. Sfintes C. V. (2010). Application of honey bee mating optimization algorithm to load profile clustering, in 2010 IEEE International Conference on Computational Intelligence for Measurement Systems and Applications (Taranto: IEEE), 113–118.

[B55] Geem Z. W. (2000). Optimal cost design of water distribution networks using harmony search (Dissertation). Korea University.

[B56] Geem Z. W. (2006). Optimal cost design of water distribution networks using harmony search. Eng. Optim. 38, 259–277. 10.1080/03052150500467430

[B57] Geem Z. W. (ed.). (2001). Music-Inspired Harmony Search Algorithm. Berlin: Springer.

[B58] Geem Z. W. Kim J. H. Loganathan G. V. (2001). A new heuristic optimization algorithm: harmony search. Simulation 76, 60–68. 10.1177/003754970107600201

[B59] Geem Z. W. Lee K. S. Park Y. (2005). Application of harmony search to vehicle routing. Am. J. Appl. Sci. 2, 1552–1557. 10.3844/ajassp.2005.1552.1557

[B60] Gholizadeh S. Barzegar A. (2013). Shape optimization of structures for frequency constraints by sequential harmony search algorithm. Eng. Optim. 45, 627–646. 10.1080/0305215X.2012.704028

[B61] Glass C. W. Oganov A. R. Hansen N. (2006). USPEX—Evolutionary crystal structure prediction. Comput. Phys. Commun. 175, 713–720. 10.1016/j.cpc.2006.07.020

[B62] Goh C. K. Tan K. C. Liu D. Chiam S. C. (2010). A competitive and cooperative co-evolutionary approach to multi-objective particle swarm optimization algorithm design. Eur. J. Operat. Res. 202, 42–54. 10.1016/j.ejor.2009.05.005

[B63] Grüninger T. Wallace D. (1996). Multimodal optimization using genetic algorithms (Master's Thesis). Stuttgart University.

[B64] Guangneng F. Lixia H. Xueguang H. (2005). Synthesis of single-crystal BaTiO3 nanoparticles via a one-step sol-precipitation route. J. Cryst. Growth 279, 489–493. 10.1016/j.jcrysgro.2005.02.054

[B65] Haddad O. B. Afshar A. Mariño M. A. (2006). Honey-bees mating optimization (HBMO) algorithm: a new heuristic approach for water resources optimization. Water Res. Manag. 20, 661–680. 10.1007/s11269-005-9001-3

[B66] Hadwan M. Ayob M. Sabar N. R. Qu R. (2013). A harmony search algorithm for nurse rostering problems. Inform. Sci. 233, 126–140. 10.1016/j.ins.2012.12.025

[B67] Hassan R. Cohanim B. de Weck O. Venter G. (2005). A comparison of particle swarm optimization and the genetic algorithm, in Proceedings of the 46thAIAA/ASME/ASCE/AHS/ASC Structures, Structural Dy-namics and Materials Conference (Austin, TX).

[B68] Heppner F. Grenander U. (1990). A stochastic nonlinear model for coordinated bird flocks, in The Ubiquity of Chaos, eds Krasner S. (AAAS Publications), 233–238.

[B69] Hoang D. C. Yadav P. Kumar R. Panda S. K. (2014). Real-time implementation of a harmony search algorithm-based clustering protocol for energy-efficient wireless sensor networks. IEEE Trans. Industr. Inform. 10, 774–783. 10.1109/TII.2013.2273739

[B70] Holland J. H. (1992). Adaptation in Natural and Artificial Systems: An Introductory Analysis with Applications to Biology, Control, and Artificial Intelligence. MIT press.

[B71] Hutter J. Luethi H. P. Diederich F. (1994). Structures and vibrational frequencies of the carbon molecules C2-C18 calculated by density functional theory. J. Am. Chem. Soc. 116, 750–756. 10.1021/ja00081a041

[B72] Hutter J. Lüthi H. P. (1994). The molecular structure of C_6_: a theoretical investigation. J. Chem. Phys. 101, 2213–2216. 10.1063/1.467661

[B73] Inderscience (2010). Cuckoo Designs Spring. Retrieved from: Alphagalileo.org

[B74] Jahanshahi G. Haddad O. B. (2008). Honey-bee mating optimization (HBMO) algorithm for optimal design of water distribution systems, in World Environmental and Water Resources Congress 2008: Ahupua'A, (Honolulu, HI). 1–16.

[B75] Karaboga D. Basturk B. (2007). A powerful and efficient algorithm for numerical function optimization: artificial bee colony (ABC) algorithm. J. Glob. Optim. 39, 459–471. 10.1007/s10898-007-9149-x

[B76] Karaboga D. Basturk B. (2008). On the performance of artificial bee colony (ABC) algorithm. Appl. Soft Comput. 8, 687–697. 10.1016/j.asoc.2007.05.007

[B77] Kennedy J. (1997). The particle swarm: social adaptation of knowledge, in Proceedings of 1997 IEEE International Conference on Evolutionary Computation (ICEC'97) (Indianapolis, IN: IEEE), 303–308.

[B78] Kennedy J. Eberhart R. (1995). Particle swarm optimization (PSO), in: Proceedings of IEEE International Conference on Neural Networks (Perth, WA: IEEE), 1942–1948.

[B79] Kennedy J. Eberhart R. C. (1999). The particle swarm: social adaptation in information-processing systems, in New Ideas in Optimization (McGraw-Hill Ltd.), 379–388.

[B80] Khan A. Sadeequllah M. (2010). Rank based particle swarm optimization, in International Conference on Swarm Intelligence (Berlin; Heidelberg: Springer), 275–286. 10.1007/978-3-642-15461-4_24

[B81] Kiranyaz S. Pulkkinen J. Gabbouj M. (2011). Multi-dimensional PSO for dynamic environments, in International Conference on Innovations in Information Technology (Tampere), 2212–2223. 10.1016/j.eswa.2010.08.009

[B82] Kirkpatrick S. Gelatt C. D. Vecchi M. P. (1983). Optimization by simulated annealing. Science 220, 671–680. 10.1126/science.220.4598.671 17813860

[B83] Koinuma H. Horiuchi T. Inomata K. Ha H.-K. Nakajima K. Chaudhary K. (1996). Synthesis of carbon clusters and thin films by low temperature plasma chemical vapor deposition under atmospheric pressure. Pure Appl. Chem. 68, 1151–1154. 10.1351/pac199668051151

[B84] Korošec P. Šilc J. Filipič B. (2012). The differential ant-stigmergy algorithm. Inform. Sci. 192, 82–97. 10.1016/j.ins.2010.05.002

[B85] Kroto H. McKay K. (1988). The formation of quasi-icosahedral spiral shell carbon particles. Nature 331:328 10.1038/331328a0

[B86] Krug M. Nguang S. K. Wu J. Shen J. (2010). GA-based model predictive control of boiler-turbine systems. Int. J. Innov. Comput. Inf. Control 6, 5237–5248. Available online at: http://www.ijicic.org/09-0646-1.pdf

[B87] Lee C. Yang W. Parr R. G. (1988). Development of the Colle-Salvetti correlation-energy formula into a functional of the electron density. Phys. Rev. B 37:785 10.1103/PhysRevB.37.785 9944570

[B88] Li G. Niu P. Xiao X. (2012). Development and investigation of efficient artificial bee colony algorithm for numerical function optimization. Appl. Soft Comput. 12, 320–332. 10.1016/j.asoc.2011.08.040

[B89] Li H.-Q. Li L. (2007). A novel hybrid particle swarm optimization algorithm combined with harmony search for high dimensional optimization problems, in Intelligent Pervasive Computing, IPC. The International Conference on: IEEE (Jeju City: IEEE), 94–97.

[B90] Li M. Lin D. Kou J. (2012). A hybrid niching PSO enhanced with recombination-replacement crowding strategy for multimodal function optimization. Appl. Soft Comput. 12, 975–987. 10.1016/j.asoc.2011.11.032

[B91] Li X. (2007). A multimodal particle swarm optimizer based on fitness Euclidean-distance ratio, in Proceedings of the 9th Annual Conference on Genetic and Evolutionary Computation: ACM (New York, NY), 78–85. 10.1145/1276958.1276970

[B92] Li-Ping Z. Huan-Jun Y. Shang-Xu H. (2005). Optimal choice of parameters for particle swarm optimization. J. Zhejiang Univ. Sci. A 6, 528–534. 10.1631/jzus.2005.A0528

[B93] Liu B. Wang L. Jin Y.-H. (2007). An effective PSO-based memetic algorithm for flow shop scheduling. IEEE Trans. Syst. Man Cybern. B 37, 18–27. 10.1109/TSMCB.2006.883272 17278555

[B94] Liu D. Tan K. C. Goh C. K. Ho W. K. (2007). A multiobjective memetic algorithm based on particle swarm optimization. IEEE Trans. Syst. Man Cybern. B 37, 42–50. 10.1109/TSMCB.2006.883270 17278557

[B95] Liu X. Liu H. Duan H. (2007). Particle swarm optimization based on dynamic niche technology with applications to conceptual design. Adv. Eng. Softw. 38, 668–676. 10.1016/j.advengsoft.2006.10.009

[B96] Liu Z. Wang S. (2006). Hybrid particle swarm optimization for permutation flow shop scheduling, in Intelligent Control and Automation, WCICA 2006. The Sixth World Congress on: IEEE (Dalian: IEEE), 3245–3249.

[B97] Ma Q. Lei X. Zhang Q. (2009). Mobile robot path planning with complex constraints based on the second-order oscillating particle swarm optimization algorithm, in Computer Science and Information Engineering, WRI World Congress on: IEEE (Los Angeles, CA: IEEE), 244–248.

[B98] Manjarres D. Landa-Torres I. Gil-Lopez S. Del Ser J. Bilbao M. N. Salcedo-Sanz S. (2013). A survey on applications of the harmony search algorithm. Eng. Appl. Artif. Intell. 26, 1818–1831. 10.1016/j.engappai.2013.05.008

[B99] Marinaki M. Marinakis Y. Zopounidis C. (2010). Honey bees mating optimization algorithm for financial classification problems. Appl. Soft Comput. 10, 806–812. 10.1016/j.asoc.2009.09.010

[B100] Marinakis Y. Marinaki M. (2009). A hybrid honey bees mating optimization algorithm for the probabilistic traveling salesman problem, in 2009 IEEE Congress on Evolutionary Computation: IEEE (Trondheim: IEEE), 1762–1769.

[B101] Martin J. François J.-P. Gijbels R. (1993). The impact of quantum chemical methods on the interpretation of molecular spectra of carbon clusters. J. Mol. Struct. 294, 21–24. 10.1016/0022-2860(93)80305-F

[B102] Martin J. M. Taylor P. R. (1996). Structure and vibrations of small carbon clusters from coupled-cluster calculations. J. Phys. Chem. 100, 6047–6056. 10.1021/jp952471r

[B103] Martonák R. Laio A. Bernasconi M. Ceriani C. Raiteri P. Zipoli F. (2005). Simulation of structural phase transitions by metadynamics. Z. Kristallogr. Cryst. Mater. 220, 489–498. 10.1524/zkri.220.5.489.65078

[B104] Martonák R. Laio A. Parrinello M. (2003). Predicting crystal structures: the Parrinello-Rahman method revisited. Phys. Rev. Lett. 9:075503 10.1103/PhysRevLett.90.075503 12633242

[B105] Millonas M. M. (1993). Swarms, Phase Transitions, and Collective Intelligence (Paper 1); and a Nonequilibrium Statistical Field Theory of Swarms and Other Spatially Extended Complex Systems (Paper 2) (No. 93-06-039).

[B106] Mitikiri P. Jana G. Sural S. Chattaraj P. K. (2018). A machine learning technique toward generating minimum energy structures of small boron clusters. Int. J. Quantum Chem. 118:e25672 10.1002/qua.25672

[B107] Mujica A. Needs R. (1997). Erratum: theoretical study of the high-pressure phase stability of GaP, InP, and InAs. Phys. Rev. B 56:12653 10.1103/PhysRevB.56.12653

[B108] Nasrinpour H. Bavani A. Teshnehlab M. (2017). Grouped bees algorithm: a grouped version of the bees algorithm. Computers 6:5 10.3390/computers6010005

[B109] Nayeem A. Vila J. Scheraga H. A. (1991). A comparative study of the simulated-annealing and Monte Carlo-with-minimization approaches to the minimum-energy structures of polypeptides:[Met]-enkephalin. J. Comput. Chem. 12, 594–605. 10.1002/jcc.540120509

[B110] Nekooei K. Farsangi M. M. Nezamabadi-Pour H. Lee K. Y. (2013). An improved multi-objective harmony search for optimal placement of DGs in distribution systems. IEEE Trans. Smart Grid 4, 557–567. 10.1109/TSG.2012.2237420

[B111] Nickabadi A. Ebadzadeh M. M. Safabakhsh R. (2008). DNPSO: A dynamic niching particle swarm optimizer for multi-modal optimization, in Evolutionary Computation, 2008. CEC 2008 (Hong Kong: IEEE), 26–32.

[B112] Oganov A. R. Glass C. W. (2006). Crystal structure prediction using ab initio evolutionary techniques: principles and applications. J. Chem. Phys. 124:244704. 10.1063/1.2210932 16821993

[B113] Omkar S. Senthilnath J. Khandelwal R. Naik G. N. Gopalakrishnan S. (2011). Artificial Bee Colony (ABC) for multi-objective design optimization of composite structures. Appl. Soft Comput. 11, 489–499. 10.1016/j.asoc.2009.12.008

[B114] Özcan E. Yilmaz M. (2007). Particle swarms for multimodal optimization, in International Conference on Adaptive and Natural Computing Algorithms (Berlin; Heidelberg: Springer), 366–375.

[B115] Pannetier J. Bassas-Alsina J. Rodriguez-Carvajal J. Caignaert V. (1990). Prediction of crystal structures from crystal chemistry rules by simulated annealing. Nature 346, 343–345. 10.1038/346343a0

[B116] Payne R. B. Sorensen M. D. (2005). The Cuckoos. New York, NY: Oxford University Press.

[B117] Pedersen M. E. H. (2010). Good Parameters for Particle Swarm Optimization. Technical Report HL1001, Hvass Lab, Copenhagen.

[B118] Petalas Y. G. Parsopoulos K. E. Papageorgiou E. I. Groumpos P. P. Vrahatis M. N. (2007). Enhanced learning in fuzzy simulation models using memetic particle swarm optimization, in Swarm Intelligence Symposium, SIS, IEEE (Honolulu, HI: IEEE), 16–22.

[B119] Pham D. T. Castellani M. (2009). The bees algorithm: modelling foraging behaviour to solve continuous optimization problems. Proc. Inst. Mech. Eng. C 223, 2919–2938. 10.1243/09544062JMES1494

[B120] Pham D. T. Castellani M. (2014). Benchmarking and comparison of nature-inspired population-based continuous optimisation algorithms. Soft. Comput. 18, 871–903. 10.1007/s00500-013-1104-9

[B121] Pham D. T. Castellani M. (2015). A comparative study of the Bees Algorithm as a tool for function optimisation. Cogent Eng. 2:1091540 10.1080/23311916.2015.1091540

[B122] Pham D. T. Ghanbarzadeh A. Koc E. Otri S. Rahim S. Zaidi M. (2005). The Bees Algorithm. Technical Note, Manufacturing Engineering Centre, Cardiff University, UK.

[B123] Pickard C. J. Needs R. (2006). High-pressure phases of silane. Phys. Rev. Lett. 97:045504. 10.1103/PhysRevLett.97.045504 16907590

[B124] Pickard C. J. Needs R. (2008). Highly compressed ammonia forms an ionic crystal. Nat. Mat. 7, 775–779. 10.1038/nmat2261 18724375

[B125] Pickard C. J. Needs R. J. (2007). Structure of phase III of solid hydrogen. Nat. Phys. 3, 473–476. 10.1038/nphys625

[B126] Pitzer K. S. Clementi E. (1959). Large molecules in carbon vapor. J. Am. Chem. Soc. 81, 4477–4485. 10.1021/ja01526a010

[B127] Pless V. Suter H. Engels B. (1994). Ab initio study of the energy difference between the cyclic and linear forms of the C_6_ molecule. J. Chem. Phys. 101, 4042–4048. 10.1063/1.467521

[B128] Poli R. (2007). An Analysis of Publications on Particle Swarm Optimization Applications. Essex: Department of Computer Science, University of Essex.

[B129] Poli R. (2008). Analysis of the publications on the applications of particle swarm optimisation. J. Artif. Evol. Appl. 2008:685175 10.1155/2008/685175

[B130] Poli R. Langdon W. B. (2002). Foundations of Genetic Programming. Berlin: Springer.

[B131] Price K. Storn R. M. Lampinen J. A. (2006). Differential Evolution: A Practical Approach to Global Optimization. Berlin; Heidelberg: Springer 10.1007/3-540-31306-0

[B132] Qu B.-Y. Liang J. J. Suganthan P. N. (2012). Niching particle swarm optimization with local search for multi-modal optimization. Inform. Sci. 197, 131–143. 10.1016/j.ins.2012.02.011

[B133] Raghavachari K. Binkley J. (1987). Structure, stability, and fragmentation of small carbon clusters. J. Chem. Phys. 87, 2191–2197. 10.1063/1.453145

[B134] Rajasekhar A. Lynn N. Das S. Suganthan P. N. (2017). Computing with the collective intelligence of honey bees–a survey. Swarm Evol. Comput. 32, 25–48. 10.1016/j.swevo.2016.06.001

[B135] Reeves W. T. (1983). Particle systems—a technique for modeling a class of fuzzy objects. ACM Trans. Graph. 2, 91–108. 10.1145/357318.357320

[B136] Reynolds C. W. (1987). Flocks, herds and schools: a distributed behavioral model. ACM SIGGRAPH Comput. Graph. 21, 25–34. 10.1145/37402.37406

[B137] Richardson P. (2008). Bats. London: Natural History Museum.

[B138] Rocca P. Oliveri G. Massa A. (2011). Differential evolution as applied to electromagnetics. IEEE Antennas Propag. Mag. 53, 38–49. 10.1109/MAP.2011.5773566

[B139] Schutze O. Talbi E.-G. Pulido G. T. Coello C. C. Santana-Quintero L. V. (2007). A memetic PSO algorithm for scalar optimization problems, in Swarm Intelligence Symposium, SIS 2007 (Washington, DC: IEEE Computer Society), 128–134.

[B140] Shao X. Cheng L. Cai W. (2004). A dynamic lattice searching method for fast optimization of Lennard–Jones clusters. J. Comput. Chem. 25, 1693–1698. 10.1002/jcc.20096 15362126

[B141] Shao X. Yang X. Cai W. (2008). A dynamic lattice searching method with interior operation for unbiased optimization of large Lennard-Jones clusters. J. Comput. Chem. 29, 1772–1779. 10.1002/jcc.20938 18351615

[B142] Shi Y. (2001). Particle swarm optimization: developments, applications and resources, in Evolutionary Computation, Proceedings of the 2001 Congress on: IEEE (Seoul: IEEE), 81–86.

[B143] Shi Y. Eberhart R. (1998). A modified particle swarm optimizer, in Evolutionary Computation Proceedings. IEEE World Congress on Computational Intelligence. IEEE International Conference (Anchorage, AK: IEEE), 69–73.

[B144] Sivasubramani S. Swarup K. S. (2009). Multiagent based particle swarm optimization approach to economic dispatch with security constraints, in Power Systems, ICPS'09. International Conference on: IEEE (Kharagpur: IEEE), 1–6.

[B145] Storn R. (1996). On the usage of differential evolution for function optimization, in Proceedings of North American Fuzzy Information Processing (Berkeley, CA: IEEE), 519–523.

[B146] Storn R. Price K. (1997). Differential evolution–a simple and efficient heuristic for global optimization over continuous spaces. J. Glob. Optim. 11, 341–359. 10.1023/A:1008202821328

[B147] Sun C. Liang H. Li L. Liu D. (2007). Clustering with a weighted sum validity function using a niching PSO algorithm, in Networking, Sensing and Control, 2007 IEEE International Conference on: IEEE (London: IEEE), 368–373.

[B148] Sun L.-Q. Gao X.-Y. (2008). Improved chaos-particle swarm optimization algorithm for geometric constraint solving, in Computer Science and Software Engineering, International Conference on: IEEE (Hubei: IEEE), 992–995.

[B149] Talbi E.-G. (2009). Metaheuristics: From Design to Implementation. Hoboken, NJ: John Wiley & Sons, Inc.

[B150] Trelea I. C. (2003). The particle swarm optimization algorithm: convergence analysis and parameter selection. Inform. Proc. Lett. 85, 317–325. 10.1016/S0020-0190(02)00447-7

[B151] Trimarchi G. Zunger A. (2007). Global space-group optimization problem: Finding the stablest crystal structure without constraints. Phys. Rev. B 75, 104113 10.1103/PhysRevB.75.104113

[B152] Unler A. Murat A. (2010). A discrete particle swarm optimization method for feature selection in binary classification problems. Eur. J. Oper. Res. 206, 528–539. 10.1016/j.ejor.2010.02.032

[B153] Ursem R. K. (2000). Multinational GAs: multimodal optimization techniques in dynamic environments, in GECCO, Las Vegas, NV; San Francisco, CA: Morgan Kaufmann Publishers Inc, 19–26.

[B154] Van Orden A. Saykally R. J. (1998). Small carbon clusters: spectroscopy, structure, and energetics. Chem Rev. 98, 2313–2358. 10.1021/cr970086n 11543299

[B155] Wales D. J. Doye J. P. (1997). Global optimization by basin-hopping and the lowest energy structures of Lennard-Jones clusters containing up to 110 atoms. J. Phys. Chem. A 101, 5111–5116. 10.1021/jp970984n

[B156] Wang J. Liu D. Shang H. (2009). Artificial intelligence and computational intelligence, in AICI'09. International Conference (Shanghai), 139–144.

[B157] Wang L. Li L.-P. (2013). An effective differential harmony search algorithm for the solving non-convex economic load dispatch problems. Int. J. Elec. Power 44, 832–843. 10.1016/j.ijepes.2012.08.021

[B158] Wang Y. Chen P. Jin Y. (2009). Trajectory planning for an unmanned ground vehicle group using augmented particle swarm optimization in a dynamic environment, in Systems, Man and Cybernetics, SMC. IEEE International Conference on: IEEE (San Antonio, TX: IEEE), 4341–4346.

[B159] Wang Y. Li B. Weise T. Wang J. Yuan B. Tian Q. (2011). Self-adaptive learning based particle swarm optimization. Inform. Sci. 181, 4515–4538. 10.1016/j.ins.2010.07.013

[B160] Wang Z. Xing H. (2008). Dynamic-probabilistic particle swarm synergetic model: A new framework for a more in-depth understanding of particle swarm algorithms, in Evolutionary Computation, CEC 2008. (IEEE World Congress on Computational Intelligence). IEEE Congress on: IEEE (Hong Kong: IEEE, 312–321.

[B161] Watts J. D. Gauss J. Stanton J. F. Bartlett R. J. (1992). Linear and cyclic isomers of C4. A theoretical study with coupled-cluster methods and large basis sets. J. Chem. Phys. 97, 8372–8381. 10.1063/1.463407

[B162] Weltner W. Jr. Van Zee R. J. (1989). Carbon molecules, ions, and clusters. Chem. Rev. 89, 1713–1747. 10.1021/cr00098a005

[B163] Weyland D. (2015). A critical analysis of the harmony search algorithm—How not to solve sudoku. Oper. Res. Persp. 2, 97–105. 10.1016/j.orp.2015.04.001

[B164] Woodley S. Battle P. Gale J. Catlow C. A. (1999). The prediction of inorganic crystal structures using a genetic algorithm and energy minimisation. Phys. Chem. Chem. Phys. 1, 2535–2542. 10.1039/a901227c 14737313

[B165] Yang X. Yuan J. Yuan J. Mao H. (2007). A modified particle swarm optimizer with dynamic adaptation. Appl. Mat. Comput. 189, 1205–1213. 10.1016/j.amc.2006.12.045

[B166] Yang X.-S. (2010a). Nature-Inspired Metaheuristic Algorithms, 2nd Edn. Cambridge, UK: University of Cambridge; Luniver Press.

[B167] Yang X.-S. (2010b). A new metaheuristic bat-inspired algorithm, in Nature Inspired Cooperative Strategies for Optimization (NICSO 2010) (Berlin; Heidelberg: Springer), 65–74.

[B168] Yang X.-S. Deb S. (2009). Cuckoo search via Lévy flights, in World Congress on Nature & Biologically Inspired Computing (NaBIC) (Coimbatore: IEEE), 210–214.

[B169] Yeh W.-C. (2009). A two-stage discrete particle swarm optimization for the problem of multiple multi-level redundancy allocation in series systems. Expert Sys. Appl. 36, 9192–9200. 10.1016/j.eswa.2008.12.024

[B170] Yeh W.-C. Chang W.-W. Chung Y. Y. (2009). A new hybrid approach for mining breast cancer pattern using discrete particle swarm optimization and statistical method. Expert Sys. Appl. 36, 8204–8211. 10.1016/j.eswa.2008.10.004

[B171] Yin P.-Y. (2004). A discrete particle swarm algorithm for optimal polygonal approximation of digital curves. J. Vis. Commun. Image Represent. 15, 241–260. 10.1016/j.jvcir.2003.12.001

[B172] Zhan Z.-H. Zhang J. Li Y. Chung H. S. (2009). Adaptive particle swarm optimization. IEEE Trans. Syst. Man Cybern. B 39, 1362–1381. 10.1109/TSMCB.2009.2015956 19362911

[B173] Zhang J. Huang D.-S. Liu K.-H. (2007). Multi-sub-swarm particle swarm optimization algorithm for multimodal function optimization, in Evolutionary Computation, CEC, IEEE Congress on: IEEE (Singapore: IEEE), 3215–3220.

[B174] Zhang J. Xie L. Wang S. (2006). Particle swarm for the dynamic optimization of biochemical processes. Comp. Aided Chem. Eng. 21, 497–502. 10.1016/S1570-7946(06)80094-5

[B175] Zhang R. Wang D. (2008). Forecasting annual electricity demand using BP neural network based on three sub-swarms PSO, in Control and Decision Conference, CCDC 2008 (Yantai: IEEE), 1409–1413.

[B176] Zhao S.-Z. Liang J. J. Suganthan P. N. Tasgetiren M. F. (2008). Dynamic multi-swarm particle swarm optimizer with local search for large scale global optimization, in Evolutionary Computation, CEC. (IEEE World Congress on Computational Intelligence). IEEE Congress on: IEEE (Hong Kong: IEEE), 3845–3852.

[B177] Zheng S.-F. Hu S.-L. Su S.-X. Lin C.-F. Lai X.-W. (2007). A modified particle swarm optimization algorithm and application, in International Conference on Machine Learning and Cybernetics (Guangzhou: IEEE), 945–951.

[B178] Zhi-Jie L. Xiang-Dong L. Xiao-Dong D. Cun-Rui W. (2009). An improved particle swarm algorithm for search optimization, in WRI Global Congress on Intelligent Systems (Xiamen: IEEE), 154–158.

[B179] Zlochin M. Birattari M. Meuleau N. Dorigo M. (2004). Model-based search for combinatorial optimization: a critical survey. Annal. Oper. Res. 131, 373–395. 10.1023/B:ANOR.0000039526.52305.af

