# Supplementary material for: Modified Particle Swarm Optimization Algorithms for the Generation of Stable Structures of Carbon Clusters, Cn (n = 3–6, 10)
Source: Front Chem. 2019 Jul 12;7:485. doi: 10.3389/fchem.2019.00485 (PMC6640203; doi:10.3389/fchem.2019.00485)
Supplement: Supplementary file 1 [file Data_Sheet_1.doc]

Manuscript ID: 465048

**Modified Particle Swarm Optimization Algorithms for the Generation of Stable Structures of Carbon Clusters, C_n_ (n = 3-6, 10)**

Gourhari Jana,^1^ Arka Mitra,^2^ Sudip Pan,^3^ Shamik Sural,^4,*^ and Pratim K. Chattaraj^1,5,*^

^1^Department of Chemistry and Centre for Theoretical Studies,

Indian Institute of Technology Kharagpur, 721302, India

*^2^Department of Electronics and Electrical Communication Engineering,*

Indian Institute of Technology Kharagpur, 721302, India

^3^Fachbereich Chemie, Philipps-Universität Marburg, Hans-Meerwein-Strasse 4, D-35043 Marburg, Germany

^4^Department of Computer Science and Engineering,

Indian Institute of Technology Kharagpur, 721302, India

***^5^****Department of Chemistry, Indian Institute of Technology Bombay, Powai, Mumbai-400076, India*

* Corresponding authors:  [shamik@cse.iitkgp.ac.in](mailto: shamik@cse.iitkgp.ac.in )  (SS); [pkc@chem.iitkgp.ac.in](mailto:pkc@chem.iitkgp.ac.in) (PKC) (ORCID; 0000-0002-5650-7666)

**Supplementary Information**

**Randomly chosen initializing coordinates of C_3_ cluster**

Structure No.: 1

C -0.33703155882 1.66818339369 -2.11940109121

C 2.59937139821 2.65235988055 0.888410087075

C -0.12738451523 1.11439452112 -0.54971173284

Structure No.: 2

C -1.81062977059 1.17712645153 -0.958010845999

C 1.59153878981 -2.12083116276 1.39617868757

C 0.825749207919 2.02779510838 -2.15239629971

Structure No.: 3

C 0.544826542324 -0.153972699666 0.537285939064

C 2.62375476445 1.61193872954 1.6547297099

C 2.90396441299 2.90920316496 -0.00553604215581

Structure No.: 4

C -0.412860811583 -0.543180146085 2.87172659849

C -2.1631130698 0.194151802365 1.46041535293

C 0.0539759905812 -0.507749606918 -0.249945244833

Structure No.: 5

C 2.87470688137 1.30442670029 -2.71762491863

C -2.9763556007 1.96399496987 -0.306421146538

C 1.10158392151 -1.03714127766 -0.486296081489

Structure No.: 6

C 0.28809613372 0.857009354911 2.38883480735

C -0.491110439244 -2.38466325868 0.247461040973

C 1.13607655457 0.591151730913 -1.9936759907

Structure No.: 7

C 0.174338331979 1.77476608504 0.320660614322

C -0.951755260753 -2.6570270784 -2.84543873107

C 1.76638140431 2.62888881075 -0.890639644464

Structure No.: 8

C 1.98979436874 0.292391795271 2.41891491875

C 0.384046741788 -1.80005803637 -1.59858604456

C 0.537308171259 2.68291451073 2.46306932659

Structure No.: 9

C 0.346183811573 -2.47649221094 1.80352754676

C -1.81789226343 2.90964921258 2.34597951366

C -2.21049364594 -1.66565779196 1.32469792612

Structure No.: 10

C -0.267344561073 -1.69690499437 0.718033952464

C -2.76337528119 -1.81167224137 -1.23764826827

C 1.60027158451 1.21172449325 -0.380174610715

**Geometric single point coordinates obtained from PSO code generation**

C -0.491706938332 1.52863765112 -0.497779371253

C 2.02169394688 1.02986372506 -0.187062364667

C 0.764958495807 1.27824943377 -0.343179012643

**Geometry optimized coordinate obtained from G09 after run by taking the final position from PSO result**

C 0.00000000 0.00000000 -1.29060700

C 0.00000000 0.00000000 1.29062100

C 0.00000000 0.00000000 -0.00001400

**Randomly chosen initializing coordinates of C_4_ cluster**

Structure No.: 1

C 0.275214716943 0.61001054277 -0.636049170555

C -1.87313840252 2.58712170312 1.29202529077

C 1.69907425149 0.296729684941 -2.48394453073

C 2.51671269384 -2.35379187047 1.05455824415

Structure No.: 2

C -1.90715486225 -1.57171873204 1.44016646951

C -0.150951391082 -2.65170025605 2.16114611074

C -0.959613738268 1.77602330653 0.30989308451

C 1.25617697482 -0.116557066079 -0.214163307695

Structure No.: 3

C -0.997822120779 -1.98647374821 -2.8268748888

C -1.42664201517 -1.7252159785 0.393470895282

C -2.20094180796 1.72079770653 -2.9454286188

C 1.06909861862 -1.31650645894 0.620842799739

Structure No.: 4

C 1.33621833913 -1.75939236992 2.09607671035

C 1.27578818986 -2.35593896197 -1.90059632399

C -0.273314195221 -0.185974349948 1.75678844024

C -0.655837939688 -1.07193335058 2.92002010506

Structure No.: 5

C 0.906761247738 -0.063972140932 2.06409120194

C 1.99661825268 1.11769339153 2.22455915226

C -2.73378453453 1.29375659946 0.47530731536

C -0.718280982686 0.157354777715 2.90057521448

Structure No.: 6

C -2.8479240956 0.197600654617 2.98310051062

C 1.84433870382 0.851275808645 -0.520995747093

C 0.304475387205 -0.884895236536 1.5276325326

C 1.28418549836 -0.615426998011 2.51528053359

Structure No.: 7

C 0.302413925763 2.51078336222 1.63860378223

C 0.138559375526 1.11576443405 -0.522069069062

C -2.7817180815 2.53410363074 0.930680061525

C -0.104669474442 1.34411529865 -2.74955021336

Structure No.: 8

C 2.60113463898 -0.822700695561 -1.3407469081

C -1.9686569403 -0.93476845925 -1.2833085757

C 2.22360664897 1.40361939942 1.84403725219

C -2.93212098639 -1.99288202605 0.311069106541

Structure No.: 9

C 0.653479604892 -2.59293477286 -1.84440038817

C 0.661457884014 1.07945681793 -2.48250094164

C -1.28893242506 0.200996796447 2.13309206268

C -0.466299762498 -0.669785513449 -2.24836392371

Structure No.: 10

C -2.22052063644 -2.03200916172 0.759059593839

C -2.88411681027 -0.259294235613 -2.52919295899

C -2.40942204731 -2.1498527563 0.608618773097

C 0.662310286268 -1.72803244845 0.379819941696

**Geometric single point coordinates obtained from PSO code generation**

**Singlet Spin Multiplicity**

**Linear**

C 0.713138639805 -0.384552293305 1.82252041526

C 1.54100552653 -0.0856614666589 0.835641399668

C -1.56854229014 0.326085621403 1.11618963637

C -0.541853468997 0.0378902572834 1.87826883074

**Ring**

C -0.72832301 0.64531435 0.55317727

C 0.26580096 0.22659825 -0.55755473

C -1.27698793 0.17513782 -0.63964323

C -0.72611959 -0.66627721 -1.55036904

**Geometry optimized coordinate obtained from G09 after run by taking the final position from PSO result**

**Linear**

C -0.64619800 0.00012400 -0.00009800

C -1.95585100 0.00016600 0.00006100

C 1.95586000 0.00045200 0.00002500

C 0.64618900 -0.00074100 0.00001200

**Ring**

C 1.23736900 -0.00041500 0.00021300

C 0.00061000 0.74920800 -0.00021300

C -0.00071200 -0.74917900 -0.00021300

C -1.23726700 0.00038600 0.00021300

**Triplet Spin Multiplicity**

**Linear**

C 0.644471000 0.000751000 0.000003000

C -0.644508000 0.000865000 -0.000003000

C 1.953048000 -0.000789000 -0.000001000

C -1.953011000 -0.000827000 0.000001000

**Randomly chosen initializing coordinates of C_5_ cluster**

Structure No.: 1

C 0.517129543231 -2.35021458901 0.818971910717

C 2.03295639395 2.11434611539 -2.84259116642

C 1.28016896771 0.0412977757473 -1.65657723247

C 2.00249789492 -2.83771290882 -2.26627937437

C 0.604379409853 -1.59963683338 -0.762558674397

Structure No.: 2

C 2.80677740478 -2.89507563884 0.794794161685

C 2.9266686793 -0.607812897543 -2.76797366693

C 1.42888051215 -2.43470010532 -0.975765718576

C 1.15109742952 2.93701586126 1.03137863855

C -0.121960743976 0.786209880292 -1.19300080918

Structure No.: 3

C 0.607559066703 -0.50728292052 -1.16637321341

C 2.94871687683 2.67065783363 0.422550387057

C 1.29836213939 2.84545343365 1.56942966402

C -0.725491186063 0.427130759752 2.59992678347

C -2.01171098309 -1.24258708724 1.63696505194

Structure No.: 4

C 2.21120149672 -0.667102806831 2.31184706722

C -1.03228848349 2.8151448888 2.69550071443

C -2.12226469363 -2.92509521816 -0.840801768974

C 1.91950910542 0.661185827698 1.09172696104

C -1.90356149424 0.585062086189 2.19696270761

Structure No.: 5

C 1.30003520609 0.829697275953 -2.76838684191

C -1.03330106685 -1.28371577332 -2.38814412358

C -1.0599228624 -0.0181623379331 1.29687480086

C 2.76797614771 0.757447303836 0.186393590015

C 1.45513496061 1.09321152839 1.72871380451

Structure No.: 6

C 2.6665529841 -2.38109575022 -0.630890586851

C -1.33998744937 -1.45765602909 -0.851540273658

C -0.885054404447 -1.66695674423 2.44675032363

C 0.848372009792 -2.11396183453 -2.9648698992

C 1.86163474605 0.838724925879 2.90534641011

Structure No.: 7

C 0.144225555929 -2.35068761277 -2.62408284167

C -1.67805375451 1.52222654413 -1.43784633059

C -0.00226774778983 0.665317664527 0.792575848666

C 2.78785744927 0.7019915926 -1.9599986068

C 2.10345117329 1.36166339837 -1.53101174975

Structure No.: 8

C 2.60941373379 2.14105510433 1.40545014145

C -0.716776988458 -2.30527456056 1.35126323513

C -2.5482140899 -1.91259800207 0.268897739553

C -0.671745453535 2.66643588554 -2.93807095098

C -0.807246440705 1.20112781059 -2.16515266408

Structure No.: 9

C 1.86611810725 0.500313093268 1.57490663756

C -1.26883105898 0.391515183064 0.123007287703

C -0.479084350423 1.57261297582 0.949575840894

C 2.14920593473 0.403081569739 1.88514700021

C -1.05158562475 -1.96311212081 -2.68397630254

Structure No.: 10

C -1.6907492779 -1.463572772 -0.143149452834

C 0.439146636638 -0.481677737827 -1.47983817515

C 1.81952661338 1.3095851763 -0.279456270803

C 0.915219954545 0.498274714503 -2.21219307872

C 2.44744371056 2.28876609588 -1.10671724477

**Geometric single point coordinates obtained from PSO code generation**

C 0.199416978696 -0.194657327995 1.08829008074

C 1.11140348185 -0.209835854103 1.96975943634

C -0.152981943015 0.111566355342 -0.13053556557

C -0.445060501224 0.756310001318 -2.52670165304

C -0.642566692528 0.38639986434 -1.28260144907

**Geometry optimized coordinate obtained from G09 after run by taking the final position from PSO result**

**Linear**

C -1.28116800 -0.00010800 -0.00014900

C 2.56692200 -0.00038500 -0.00001700

C -2.56698800 -0.00026000 0.00009900

C 0.00002500 0.00061100 -0.00001400

C 1.28120900 0.00014200 0.00008200

**Ring**

C 0.000000000 0.000000000 1.278273000

C 0.000000000 0.743725000 0.060745000

C 0.000000000 1.809930000 -0.699882000

C 0.000000000 -1.809930000 -0.699882000

C 0.000000000 -0.743725000 0.060745000

**Randomly chosen initializing coordinates of C_6_ cluster**

Structure No.: 1

C -2.4116507184 -2.34607246461 -2.85794296907

C 0.557443826881 1.77461512858 1.51828203353

C 1.4237284434 -2.12249298877 0.981748382578

C -2.42417314347 -0.681911864075 -1.71116740107

C -2.45274693135 -0.310761854811 0.344311565002

C 1.02258060099 -0.315794521641 -0.087219396254

Structure No.: 2

C 0.24155660659 2.00374909569 0.592038895298

C 2.08592004265 -2.56473301029 1.26698649552

C 0.421216358114 -0.714657012497 2.3804924493

C -2.40645919735 -2.72051285077 1.29698369773

C 0.671602811695 1.754804427 -0.381358576433

C -0.122767765629 -1.40654442051 -1.87485819766

Structure No.: 3

C 0.0387421530816 1.350000423 2.9043601302

C -2.13452059814 -0.0667894748756 -0.698814558729

C -1.64582946562 -0.434325220065 0.942197841907

C -0.0570591285098 0.948728700506 0.137594482661

C 1.83345482213 -1.23417623553 -0.607247595275

C -1.47025562246 -0.235309044989 1.51204690212

Structure No.: 4

C 1.53955138777 1.05500767165 -0.245796934377

C 0.436414541234 1.32552517022 -0.0375691561379

C 0.99257765147 -2.14343020812 -0.695070837558

C -2.07097957549 1.22149667234 -1.83818189273

C 1.9836575932 -2.81889711207 0.338917843281

C -0.421660328932 -0.416091878956 0.836020352646

Structure No.: 5

C 2.17396313568 0.830598939612 -0.797812720198

C 1.28736028344 -2.79150197321 -1.9245453649

C -1.7930331735 2.41913602078 -1.9454563853

C 2.66398839819 1.43177551606 2.44770930652

C 2.77892949965 -0.380542410375 -0.658022267817

C 1.49221346429 -2.90901783196 -1.24714085533

Structure No.: 6

C -2.25682100112 -0.486593774243 -1.7509971226

C 0.793413922667 -0.750709630306 -0.995018295471

C -1.3392755803 0.838100978722 0.213092513961

C -1.14668693125 2.0657378926 0.300366268485

C -1.67951802455 -0.418954561292 -1.36467137228

C 1.2355414154 1.20229534304 0.0802154058661

Structure No.: 7

C 1.51974396276 1.91484767197 1.76747487584

C -0.686666228901 -0.270245136461 0.878356066716

C 0.0963295731815 2.86138300804 -1.85059182503

C -1.0984137082 -2.21217540404 -1.06305082273

C -0.970388922348 -0.720167669635 -0.240195699932

C 2.63613239821 -0.886614901069 0.681337097155

Structure No.: 8

C -1.33167944603 -0.682881818348 -0.20028277055

C -0.168305960189 1.68092705475 -1.69343790589

C 0.182514344997 1.43340494555 2.06147951826

C 1.51120933928 2.29044770623 1.81202882805

C 2.67871087179 -1.9789482722 -0.545982751645

C -2.06262393639 -2.16927819904 2.49299831724

Structure No.: 9

C 2.03012632397 -2.4243805245 -0.50800616874

C 0.723162356134 -2.0235849478 -1.77095028686

C -0.40631173049 1.08513069843 -0.702888502926

C 0.63606581092 2.45562431088 -0.133692564993

C -2.08582672078 -1.34119372036 -0.555516976229

C 1.49844226087 -0.815759306709 -2.17091953382

Structure No.: 10

C 0.0350398855736 -2.70327284942 -1.60950845463

C 2.34839317826 0.430410801567 -0.41081359859

C 2.10197254282 -1.27168813143 2.68241852737

C -1.18104117643 0.817545389402 -2.77593059431

C 1.43510977719 -2.18320591425 -1.46275117781

C -0.987763162677 -0.95376106243 0.397000873962

**Geometric single point coordinates obtained from PSO code generation**

**Linear**

C -0.46678278 -0.00000000 0.04050650

C -0.85823308 0.00000000 6.50884308

C 0.00121796 0.00000000 2.72380973

C -0.26305623 -0.00000000 1.21478517

C 0.00045155 0.00000000 3.92441643

C -0.48313928 0.00000000 5.37758734

**Ring**

C -1.48407168 0.07441306 -0.89579139

C 0.62881711 -0.43281963 -0.83253738

C -0.22053303 0.93080498 0.89847577

C -1.32083570 0.81209883 0.17657252

C -0.57884497 -0.86408349 -0.91326522

C 0.86613230 -0.32617535 0.75714368

**Geometry optimized coordinate obtained from G09 after run by taking the final position from PSO result**

**Singlet Spin Multiplicity**

**Linear**

C -0.00049200 3.22368400 0.00000000

C 0.00088500 -3.22355800 0.00000000

C 0.00000000 0.63688400 0.00000000

C -0.00006200 1.92563800 0.00000000

C -0.00044400 -0.63702800 0.00000000

C 0.00011200 -1.92562000 0.00000000

**Ring**

C 1.27005800 0.73326900 0.00000000

C 0.00000000 -1.46653700 0.00000000

C -1.27005800 0.73326900 0.00000000

C 0.00000000 1.10223100 0.00000000

C 0.95456000 -0.55111600 0.00000000

C -0.95456000 -0.55111600 0.00000000

**Triplet Spin Multiplicity**

**Linear**

C 0.000000000 0.000000000 3.221835000

C 0.000000000 0.000000000 -3.221835000

C 0.000000000 0.000000000 0.636647000

C 0.000000000 0.000000000 1.922940000

C 0.000000000 0.000000000 -0.636647000

C 0.000000000 0.000000000 -1.922940000

**Randomly chosen initializing coordinates of C_10_ cluster**

Structure No.: 1

C -2.00293128556 -2.8026903843 0.130496619338

C -0.554031647961 -2.75759259662 0.68591854402

C 0.798869273938 -2.53990833908 0.929866827002

C -1.79073655956 0.587412333339 0.265141526785

C -2.52943621463 0.188320602305 0.500877316263

C -3.27181059276 -0.904562417717 0.0951487486139

C 1.21375467694 -1.33005242372 0.607186132484

C -0.184989467942 1.52909865808 -0.356170237994

C 1.00246348605 0.667170785833 0.304187947249

C 1.58122684408 0.0471053853894 0.0315010962835

Structure No.: 2

C -1.63782034002 -2.82521767955 1.07884682541

C -0.380910619532 -2.92666675873 0.548574007094

C 0.203289591056 -2.92590520954 0.329423137672

C -1.79976922725 1.26794705041 -0.206484825649

C -2.32290194102 0.416711595584 0.291185085103

C -2.59869366997 -1.13478468405 0.660653453032

C 1.61513448703 -2.1604666142 0.0713829367071

C -0.293002188235 1.51811310585 0.312581029324

C 0.564685521311 0.727140666503 -0.262973815765

C 1.70040120454 -0.0297047309379 0.400977855732

Structure No.: 3

C -1.85622203526 -2.49447140047 0.914101772292

C -0.909792761462 -2.93588229426 0.19546596077

C 0.923008579979 -2.94607880323 0.82825011599

C -1.87324847429 0.865315849509 0.350278185838

C -3.13448069083 0.367578434852 0.390484261083

C -2.8917406883 -1.19353367356 0.630600722121

C 1.06368782969 -1.49813569918 0.568875073941

C -0.237313026203 1.67761674455 0.112830569777

C 1.29792419902 0.602155285118 -0.529566698525

C 1.13919182996 -0.268825835671 0.385408195939

Structure No.: 4

C -1.96948807376 -2.1085491167 0.111752718833

C -0.705445475813 -3.07686223676 0.637369488371

C 0.736667735792 -2.96428547393 0.536886191871

C -1.68512705714 0.825123924868 0.20692350774

C -2.5968862948 -0.249831358861 0.457047086816

C -2.76560292301 -1.01083606468 0.156112341869

C 1.29625771319 -1.56709461135 0.192076767307

C -0.295913203152 1.84253512394 -0.250851250847

C 1.03737562595 0.496962577817 -0.374478935318

C 1.61801033909 -0.803990899701 0.359408773737

Structure No.: 5

C -2.00433991402 -2.01376546168 0.492455395683

C -1.03342237801 -3.04850108335 0.187930790299

C 0.0716969157511 -2.66710691733 0.159589787455

C -1.28387613575 1.44451734792 -0.321171302944

C -2.54981107654 -0.107513481301 0.340254227302

C -3.08374573552 -1.40888339955 0.75174952965

C 1.7871270467 -1.9780703732 0.0999192247877

C 0.0786082838449 1.17308884753 -0.542607179286

C 0.688264814147 0.791495950277 -0.0210577205209

C 1.89240049664 0.0922493562835 0.270366807214

Structure No.: 6

C -2.26820487474 -2.33588895437 0.757880328823

C -0.847704344773 -2.91002534703 0.300087892611

C 0.0836360273766 -2.76097417762 0.473671114193

C -1.64198156307 1.42590934421 0.425804387272

C -2.46636151072 -0.143886570192 0.0528796810144

C -2.87065361416 -1.34001277285 0.481318662838

C 1.22152210014 -2.12836922306 0.652000093909

C -0.299065505109 1.07231787987 -0.391457915058

C 1.43309694119 0.799631172731 0.304787284359

C 1.68029556687 -0.484633889926 0.39975552968

Structure No.: 7

C -2.00643414778 -2.11254478952 0.32479256742

C -1.23457293609 -3.51289944798 0.286547815358

C 0.558972171232 -2.91692043999 0.690138929423

C -1.53755142202 0.65714082326 -0.161739422163

C -2.89460475752 0.393859615727 -0.228645525534

C -3.17132758221 -1.02219106319 0.13448149573

C 1.07538692298 -2.12097509611 0.557742342244

C -0.155889306492 1.05045043144 -0.519049933973

C 0.622481051915 0.451176802974 -0.127426825344

C 1.70410223838 -0.00345061268378 -0.0139344949371

Structure No.: 8

C -2.24451419169 -2.89119760349 1.07395953647

C -0.889028483585 -3.0342955922 0.936159678176

C 0.143539621546 -2.6784587261 0.582711691867

C -2.15810255035 1.37458082596 -0.0791788196151

C -2.54058866801 0.190993817011 -0.0891382768972

C -3.15174481282 -1.53864876833 0.695531321677

C 1.06568310343 -1.60501679016 0.415928006798

C -0.37555976375 1.31341997227 -0.606311069863

C 0.745883480035 0.806372030224 0.163719288489

C 1.78097422977 -0.445380271595 -0.222759378855

Structure No.: 9

C -1.69700428809 -2.22008206427 0.616116412911

C -0.545780267811 -2.85006602586 0.774929893833

C 0.227110620982 -2.9265762564 0.422126384416

C -2.15789285281 0.571275011853 -0.0868381176473

C -2.51739097589 0.452686855783 -0.0940672039706

C -2.42733429013 -1.37401831071 0.0910473152096

C 1.82620535805 -1.3296190637 -0.173470106125

C -0.530664514221 1.77772952421 -0.391512498459

C 1.23066048684 0.67753646293 0.356122690113

C 1.93699670762 0.0692732919301 -0.428077302628

Structure No.: 10

C -2.15550480107 -2.26946423979 0.803978053184

C -0.880917520145 -3.4990785152 0.897735338539

C 0.89713565027 -3.21841827579 0.219299217338

C -1.5235906797 1.26847609983 0.0236831914276

C -2.53770222913 -0.306301734711 0.480658120517

C -2.87842458598 -1.71260995023 -0.0324654246429

C 1.9130667244 -1.91747480352 -0.0972195563911

C -0.80982417325 1.12134821652 0.238182570314

C 1.38892079754 0.537710660873 -0.518470112106

C 1.64096290075 -0.696133029921 -0.404140849472

**Geometric single point coordinates obtained from PSO code generation**

C -2.07119334111 -2.36285143159 0.515825777073

C -0.835342259281 -3.05365239292 0.52373845509

C 0.478933108399 -2.82184079445 0.479956582257

C -1.65570049081 1.00978988355 -0.0118121647258

C -2.64355910776 0.0676667754958 0.186369787896

C -2.79358495825 -1.22861314896 0.38781227362

C 1.47989819925 -1.80857341081 0.2803456198

C -0.304436806442 1.40613789057 -0.188681013711

C 0.981673873253 0.761077585649 -0.0883983571402

C 1.65753239103 -0.358251508629 0.127448228395

**Geometry optimized coordinate obtained from G09 after run by taking the final position from PSO result**

C 0.00000000 0.00000000 2.07932700

C 0.00000000 1.22227200 1.68225400

C 0.00000000 1.97776700 0.64259400

C 0.00000000 -1.97776700 -0.64259400

C 0.00000000 -1.97776700 0.64259400

C 0.00000000 -1.22227200 1.68225400

C 0.00000000 1.97776700 -0.64259400

C 0.00000000 -1.22227200 -1.68225400

C 0.00000000 0.00000000 -2.07932700

C 0.00000000 1.22227200 -1.68225400
