# Supplementary material for: Modified Particle Swarm Optimization Algorithms for the Generation of Stable Structures of Carbon Clusters, Cn (n = 3–6, 10)
Source: Front Chem. 2019 Jul 12;7:485. doi: 10.3389/fchem.2019.00485 (PMC6640203; doi:10.3389/fchem.2019.00485)
Supplement: Supplementary file 2 [file Data_Sheet_2.doc]

**Table of Content**


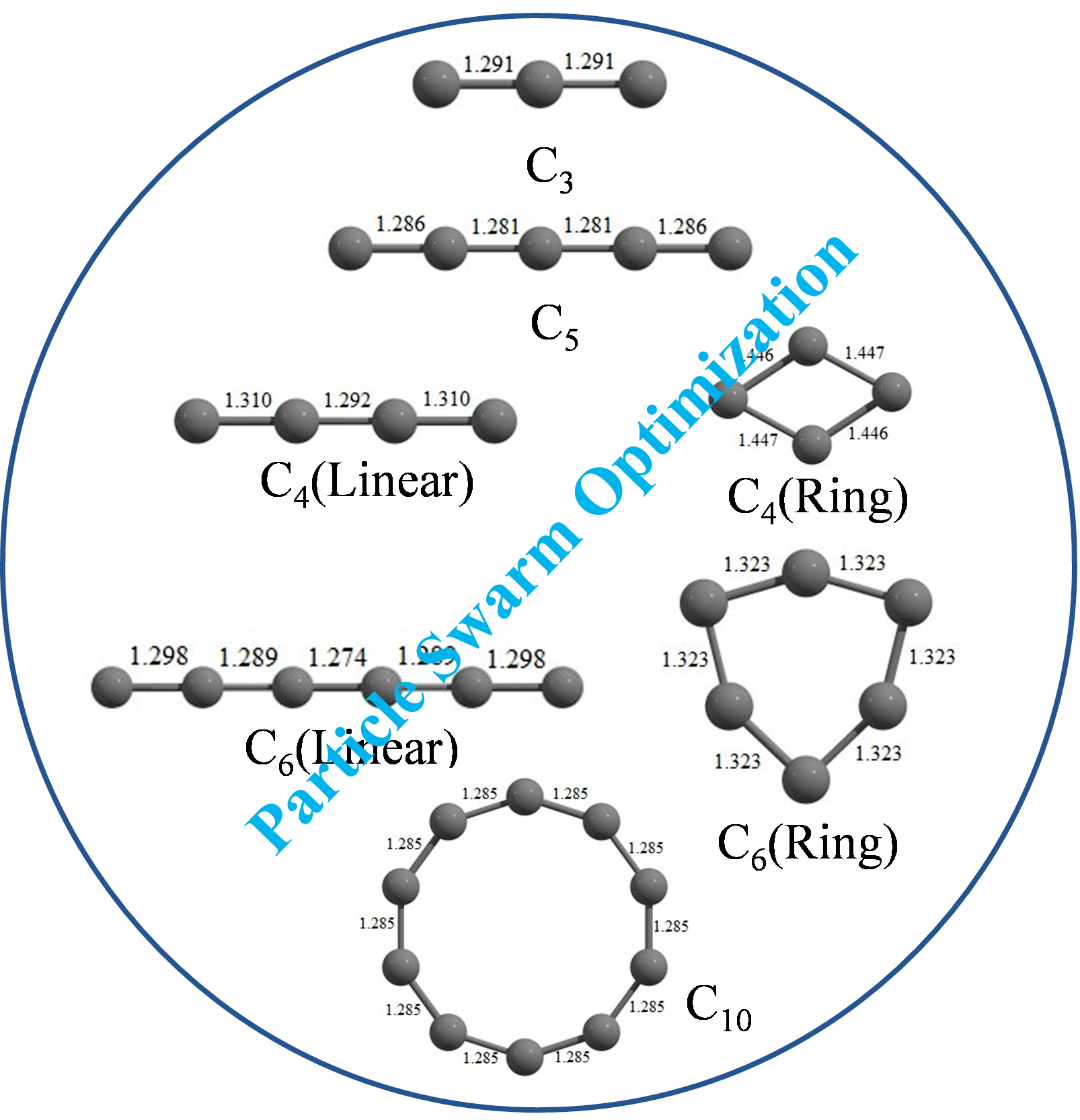


Global minimum energy configuration search using a modified Particle Swarm Optimization (PSO) algorithm in a multidimensional search space augmented by quantum chemical calculations on small carbon clusters, C_n_ (n = 3-6, 10).
